# Exogenous Heat Shock Proteins in Oncology: Biological Roles and Clinical Implications

**DOI:** 10.3390/cancers18142322

**Published:** 2026-07-18

**Authors:** Alexandra Sokolenko, Thiago Gomes Heck, Elena Mikhailova, Lilian Corrêa Costa Beber, Bruna Steffler, Mirna Stela Ludwig, Huile Gao, Maxim Shevtsov

**Affiliations:** 1 Department of Molecular Biotechnology, Saint-Petersburg State Institute of Technology, 190013 Saint Petersburg, Russia; alex_sokol_03@mail.ru; 2 Department of Life Sciences, Universidade Regional do Noroeste do Estado do Rio Grande do Sul, Ijuí 98700-000, Brazil; thiago.heck@unijui.edu.br (T.G.H.); liliantutty@hotmail.com (L.C.C.B.); bruna.steffler@sou.unijui.edu.br (B.S.); ludwig@unijui.edu.br (M.S.L.); 3 Laboratory of Biomedical Nanotechnologies, Institute of Cytology of the Russian Academy of Sciences (RAS), 194064 Saint Petersburg, Russia; mikhailovaer@yandex.ru; 4 Key Laboratory of Drug-Targeting and Drug Delivery System of the Education Ministry, Sichuan Engineering Laboratory for Plant-Sourced Drug and Sichuan Research Center for Drug Precision Industrial Technology, West China School of Pharmacy, Sichuan University, Chengdu 610064, China; gaohuile@scu.edu.cn; 5 Department of Radiation Oncology, Technishe Universität München (TUM), Klinikum Rechts der Isar, Ismaninger Str. 22, 81675 Munich, Germany

**Keywords:** heat shock proteins, HSP70, HSP90, chaperones, biomarker, circulating HSP, cancer therapy

## Abstract

Heat shock proteins (HSPs), particularly HSP70 and HSP90, are highly conserved molecular chaperones that protect cells from a wide range of stressors and maintain proteome homeostasis. In cancer, tumor cells frequently overexpress and actively release HSPs into the extracellular space and circulation in response to metabolic alterations, hypoxia, oxidative stress, and therapeutic interventions. Consequently, circulating HSP levels are often elevated in patients with malignancies compared with healthy individuals. This review summarizes current evidence on the diagnostic, prognostic, and predictive value of circulating HSPs in both solid and hematological cancers. Clinical studies indicate that circulating HSP concentrations are associated with tumor type, disease stage, lymph node involvement, metastatic burden, treatment response, and risk of recurrence. Importantly, membrane-associated and extracellular vesicle-associated forms of HSP70 appear to exhibit greater tumor specificity than freely circulating proteins, highlighting the importance of selecting appropriate analytical approaches for biomarker assessment. Beyond their utility as biomarkers, extracellular HSPs actively participate in tumor biology and anti-tumor immunity. Depending on the cellular and immunological context, they can either support tumor progression or stimulate immune responses through activation of natural killer cells, antigen-presenting cells, and cross-presentation of tumor-derived antigens. These immunomodulatory properties have provided the foundation for the development of HSP-based vaccines and adoptive immunotherapeutic strategies, several of which have demonstrated encouraging results in clinical trials. We further discuss the relationship between extracellular chaperone biology and responses to major anticancer treatments, including radiotherapy, chemotherapy, HSP90-targeted therapies, and immune checkpoint blockade. In conclusion, the available evidence supports circulating extracellular HSPs as promising non-invasive biomarkers and potential pharmacodynamic indicators that may improve patient stratification, treatment monitoring, and prediction of therapeutic efficacy in clinical oncology.

## Simple Summary

Heat shock proteins (HSPs) are stress-responsive molecules that help cells maintain their normal functions under adverse conditions. In cancer, elevated levels of HSPs can be detected in the bloodstream, making them attractive candidates for minimally invasive biomarkers. In this review, we examine the clinical and biological significance of circulating HSPs in oncology. Beyond their potential use in cancer detection, these proteins may provide valuable information on disease stage, prognosis, and treatment response. We also discuss their active involvement in tumor development, progression, and interactions with the immune system. Current evidence indicates that the diagnostic and prognostic value of circulating HSPs depends on multiple factors, including cancer type, the specific circulating form of the protein, and the patient’s immune status. A better understanding of the complex and often dual roles of HSPs is essential for accurate clinical interpretation and for the development of novel therapeutic strategies aimed at either harnessing or inhibiting their functions in cancer.

## 1. Introduction

Cancer is characterized by the uncontrolled proliferation of abnormal cells that exceed normal tissue boundaries, invade adjacent tissues, and can disseminate to distant organs through the processes of invasion and metastasis. It is the leading cause of death worldwide, accounting for one in every six deaths [1]. In the USA alone, 613,349 people died of cancer in 2023 [2]. Among the most common types are lung (2.5 million cases in 2022), breast (2.3 million cases), colon and rectum (1.9 million cases) and prostate cancers (1.5 million cases) [1]. Despite the condition’s intimidating reputation, numerous cancer types can be treated effectively if detected early.

In this perspective, the heat shock proteins (e.g., HSP90, HSP70—including its specific isoforms HSP72, HSP73, etc.—and HSP40) have emerged as valuable markers for investigation. Due to their intracellular chaperone nature, HSPs are involved in multiple antioxidant [3,4], anti-inflammatory [5,6,7] and anti-apoptotic roles [3,8,9], and are essential in keeping and restoring homeostasis. Especially in highly metabolically active cells with fast replication rates, such as the tumoral cells, HSPs are necessary to ensure proteostasis. Therefore, studies have consistently shown that they are highly expressed and released into plasma, serum, liquor, and cell culture media [10,11,12,13,14]. In extracellular space, HSPs can help shape the tumoral microenvironment and influence the intercellular network by either helping in sensitizing immune cells [15,16,17] or enhancing their tolerance to emerging cancer [18,19]. Thus, it became necessary to deeply investigate the HSPs’ ability to modulate immune defense, and how their detection can help in cancer diagnosis and prognosis under different interventional strategies.

Several reviews have addressed distinct aspects of eHSP biology, including extracellular HSP70 as a molecular biomarker and therapeutic target, the role of eHSP90 in extracellular matrix remodeling and tumor invasiveness, the immune-modulatory functions of circulating eHSPs, and broader perspectives on eHSPs in oncology. However, a comprehensive, cancer-type-specific synthesis of quantitative circulating eHSP data across diverse malignancies, integrating diagnostic, prognostic, and predictive dimensions within a unified analytical framework, has not previously been provided. In particular, prior reviews have not systematically distinguished between the four biologically distinct eHSP pools (i.e., intracellular, membrane-associated, vesicle-bound, and freely circulating) as a basis for interpreting cross-study clinical data. The present review addresses these gaps. Rather than proposing a new conceptual framework, its primary contribution lies in systematically consolidating extracellular and circulating HSP findings across individual cancer entities, a synthesis supported by constructed quantitative clinical tables that enable cross-malignancy comparison of biomarker performance.

We hereby provide a cancer-type-specific synthesis of circulating eHSP findings across major solid and hematological malignancies, framed by a four-pool compartment model. These clinical data are integrated with the biological roles of eHSPs in tumor progression, their modulation by therapeutic interventions, and critical appraisal of methodological limitations and translational challenges.

## 2. Detection of Extracellular Heat Shock Proteins in Cancer

Malignant tumors are highly associated with various types of stress, including metabolic, hypoxic and immune. In response to these conditions, tumor cells accumulate chaperone proteins inside themselves and also actively secrete them into the extracellular space. This fundamentally differentiates tumor cells from healthy ones, which normally release basal amounts of heat shock proteins during natural intercellular communication. However, physiological conditions, including acute physical exercise, low-grade systemic inflammation and thermal stress, can drastically elevate circulating HSP levels in healthy individuals, thereby introducing a potential for false-positive interpretation [20,21,22]. In the case of oncological states, the concentration of these proteins in biological fluids often exceeds the norm by 1.5–5 times, which reflects the intensity of tumor stress and the aggressiveness of the neoplasm [10,23].

Among the most clinically significant extracellular chaperones, several molecular families are distinguished. HSP70 stands out, encompassing isoforms such as HSPA1A and HSPA1B. Its level has emerged as a widely explored circulating biomarker in the field of cancer research. HSP90 comprises two cytosolic variants: constitutively expressed HSP90β and stress-induced HSP90α [20]. HSP90α predominantly undergoes secretion through non-canonical pathways. It plays a critical role in processes such as extracellular matrix remodeling, lymphangiogenesis, and cellular invasion [24,25]. Moreover, chaperone proteins originating from the endoplasmic reticulum, such as GRP78/BiP (HSPA5) and GRP94 can be found not only within cells but also on their surfaces and in systemic circulation. This phenomenon is particularly significant in situations of endoplasmic reticulum stress, which frequently occurs in rapidly growing tumors [26]. Furthermore, the presence of HSP60 and HSP27 has been detected in bodily fluids of individuals with different types of cancer [23,27].

Within this context, four biologically distinct pools of eHSPs are consistently differentiated throughout this review as follows:

1. Intracellular pool, retained within the cell;
2. Membrane-associated pool, located on the surface of the tumor cell as a prerequisite for active secretion;
3. Vesicle-associated (exosomal or/and microvesicular) pool, released via active, energy-dependent secretion specifically by malignant cells;
4. Freely circulating soluble pool in serum or plasma, which can also arise from passive release by dying or damaged cells irrespective of malignancy.

The membrane- and vesicular-associated pools confer a fundamentally tumor-specific character on circulating eHSPs, whereas the freely soluble pool is comparatively less specific [28,29]. Clinical data nonetheless suggest a correlation between the overall level of circulating eHSPs and disease severity, lymph node involvement, and histological differentiation, as well as the risk of early relapse [10,11,12].

Nonetheless, circulating eHSP profiles are not universal and demonstrate pronounced nosological specificity both in terms of qualitative composition of the dominant isoforms and their quantitative levels. These differences are conditioned by a variety of factors: the histological origin of the tumor, the features of its microenvironment, the degree of vascularization and hypoxia, the stage of the disease, and the role of various cellular sources (i.e., tumor, immune, and stromal) in the formation of the circulating pool [23,27,30]. Combined, these factors may give rise to cancer-specific patterns of circulating heat shock protein levels—a concept explored throughout this review, though, as discussed in Section 3.3 and Section 6, current evidence supports this as a provisional rather than firmly established model.

In the following subsections we will systematically analyze these patterns in major solid and hematological malignancies, assessing their diagnostic, prognostic and predictive significance.

## 3. Cancer-Type-Specific Profiles of Circulating eHSPs

### 3.1. Solid Tumors

#### 3.1.1. Brain Tumors

Brain tumors pose a particular challenge for circulating biomarker development, since the blood–brain barrier (BBB) critically limits the release of tumor-derived proteins into systemic circulation even with partial BBB dysfunction, as observed, for instance, in high-grade gliomas [31]. Nonetheless, circulating heat shock proteins, particularly HSP70, can be detected in plasma and serum of patients with primary tumors at concentrations that exceed those in healthy individuals, and their levels correlate with both diagnostic and prognostic characteristics of the disease [11]. Among all primary brain tumors, glioblastoma (WHO grade IV) has been studied most thoroughly. The vesicular fraction, which reflects the active secretion of cells expressing HSP70, is associated with the degree of malignancy of the tumor: in glioblastoma, this fraction is significantly higher compared to both healthy individuals and lower-grade tumors, whereas in grade III oligodendroglioma and astrocytoma it does not critically differ from control values (see Table 1) [11]. Furthermore, free eHSP70 is also detectable in serum, with a significant difference observed solely in glioblastoma samples in clinical study—attributable to higher membrane HSP70 density in grade IV versus grade III tumors, as discussed further below [11,32].

The prognostic significance of eHSP70 also depends on tumor grade, reflecting an underlying immunological dichotomy. In glioblastoma, a high level of vesicular eHSP70 correlates with a reduced CD4+ T helper count and poor overall survival [11]. This has been interpreted as reflecting tumor-microenvironment-driven immunosuppression with reduced NK cell efficacy, although the source study itself frames this interpretation as provisional [11]. In contrast, in grade III gliomas, an increased level of vesicular eHSP70 is associated with an increased frequency of activated NK cells and with a more favorable survival prognosis. This paradox is resolved by differences in immune competence of the host: when the CD4+ compartment remains intact, vesicular eHSP70 can exert its immunostimulatory potential [11,32], whereas in glioblastoma the collapse of this compartment renders this signal functionally ineffective [11,32].

**Interpreting eHSP70 in CNS Tumors: Passive Release and Confounding Factors**

Correctly interpreting circulating eHSP70 in brain tumors requires considering four key aspects related to the central nervous system (CNS) tumor biology, with direct implications for clinical practice. Foremost, vesicular eHSP70 is a more informative marker than its free counterpart, since partial BBB dysfunction in tumors preferentially permits microvesicle passage into the bloodstream rather than free proteins [11]. Second, the absence of a considerable HSP70 increase in blood in grade III glioma, despite its presence in the tumor tissue, likely reflects relatively intact BBB integrity, smaller tumor size, and lower surface HSP70 density, all limiting detectable systemic vesiculation at this stage [11,32]. Third, the immunostimulatory effect of vesicular eHSP70 (exerted through NK cell activation) is substantially modulated by the immunosuppressive environment of the brain tumor, making host immune status a key determinant of interpretation [11]. Fourth, and critically for clinical practice, serum eHSP70 also reflects passive release from necrotic cells: Werner et al. demonstrated markedly elevated free eHSP70 in glioblastoma patients with extensive tumor necrosis [33], meaning a single elevated measurement cannot by itself distinguish an actively secreting aggressive tumor from extensive necrosis—which, during treatment monitoring, may instead indicate a favorable therapeutic response. Because the vesicle-bound/exosomal fraction specifically captures active cellular secretion while remaining comparatively unconfounded by passive necrotic release, isolating this fraction rather than relying on total or free-soluble eHSP70 is essential for correct clinical interpretation. We recommend that future validation protocols employ dual isolation methods (combining affinity capture or ultracentrifugation for the vesicular fraction with whole-serum ELISA) to formally distinguish these biological sources [11,33]. Table 1 shows the quantitative parameters based on which the relevant conclusions were drawn.

**Other Members of HSP Family in Gliomas**

Beyond the HSP70 family, other proteome and transcriptome studies also demonstrate that other chaperone proteins play a crucial role in the progression of gliomas. GRP78/BiP (HSPA5) and GRP94 (HSP90B1) are significantly elevated in glioblastoma compared to normal brain tissue and early stages of gliomas, indicating an association with tumor progression [34], and HSPB1 or HSP27 is a highly significant predictor in the context of patient survival [34]. Notably, in glioblastoma-derived cell lines the expression of the chaperones differs from that observed in the primary clinical samples [35]: HSP70 levels remain relatively low, despite the high aggressiveness of these cells, likely because the culture lacks hypoxic and necrotic stimuli present in vivo [35]. This underscores the importance of clinical-sample-based research for a deeper understanding of chaperone biology.

#### 3.1.2. Circulating HSPs in Breast Cancer: Levels, Correlations, and Prognostic Implications

Among oncological entities, breast cancer in the context of circulating HSPs occupies a special place for two reasons. First, it was the first implementation of plasma HSP vesicular fraction for serial monitoring during treatment. Second, plasma HSP90α not only serves as a diagnostic marker but also as an active participant in the process of lymph node metastasis, having been tested in a registered clinical trial [12,36].

Increased concentrations of serum HSP70 are associated not only with the presence of the tumor, but also with its histological grade of malignancy and the Ki-67 proliferation index [12] (see Table 2). It should be noted, however, that the available evidence primarily reflects correlations with markers of tumor aggressiveness rather than early-stage disease detection, and the utility of serum eHSP70 as a screening tool in early breast cancer remains to be prospectively validated [12,37].

Complementary evidence from tissue-based studies further contextualizes the circulating data. Research by Dimas and colleagues reported the results of 23 studies [38]. Collectively, it confirmed that HSP70 and HSP90 can serve as harbingers of the progression of breast cancer. However, the majority of studies in this meta-analysis employed immunohistochemistry, which quantifies intracellular protein levels within the tumor parenchyma rather than in the circulation. Intracellular overexpression of HSP70 does not automatically translate into equivalent rates of extracellular vesicular secretion, as expression of mHSP70 is governed by distinct biological mechanisms including lipid raft association and metabolic stress signaling, which are regulated independently from cytoplasmic chaperone levels [28,29]. Consequently, the observed association between high intratumoral HSP70 and estrogen receptor status [38] provides mechanistic context for circulating data but cannot be directly extrapolated to plasma or serum measurements without paired validation.

Plasma HSP90α is a unique marker that complements information about breast cancer beyond what HSP70 provides. Unlike CEA and CA153, plasma HSP90α levels have a direct relationship with lymph node involvement [25,36]. The mechanism of action of HSP90α was revealed during in vitro and in vivo experiments: the protein interacts with the LRP1 receptor located on the endothelial cells of lymphatic vessels, which leads to activation of the AKT pathway and increased secretion of CXCL8. This, in turn, stimulates the growth of lymphatic vessels [25]. In experimental animal models where the neutralization of HSP90α was carried out with the help of antibodies, there was a significant decrease in the density of lymphatic vessels in the primary tumor and a decrease in the frequency of metastasis to sentinel lymph nodes [25]. Nomogram-based analysis further demonstrated that plasma HSP90AA1 levels do not correlate with traditional markers [36,39]. However, the specificity of this protein remains an open question, as in some cases not related to cancer, such as wound healing and hypoxia, plasma HSP90α levels also increase.

**Table 2:** Circulating eHSP levels in breast cancer patients and healthy controls.

eHSP	Sample Type	Biomarker Level in Cancer	Level in Controls	Source
Circulating eHSPs (serum, plasma)				
HSP70 (HSPA1A)	Serum	Median 1037 pg/mL	300 pg/mL	[12]
HSP70	Serum	Elevated (98.3% positive)	Lower	[37]
HSP90α	Plasma	Elevated; ↑ with N stage	Lower	[25]
HSP90α	Plasma	Independent predictor of onset and metastasis	–	[36]
HSP90α (pan-cancer)	Plasma	Median 157.8 ng/mL	<69.19 ng/mL cutoff	[39]
Tissue HSPs				
HSP70/HSP90	Tissue	HSP70 high → worse DFS; HSP90 high → worse OS	-	[38]

Abbreviations: DFS, disease-free survival; OS, overall survival; ↑, increased; →, associated with.

#### 3.1.3. HSPs in Non-Small Cell Lung Cancer (NSCLC): From Gross Tumor Volume to Stage Stratification

Among solid malignancies, non-small cell lung cancer (NSCLC) represents one of the most thoroughly characterized contexts for circulating HSPs, with independent cohort evidence for eHSP70, HSP27, and HSP90α as liquid biopsy candidates [29,40,41,42]. For eHSP70 in particular, two features directly shape its clinical interpretation: a pronounced stage-dependent profile and a sensitivity to host immune competence.

Circulating plasma HSP70 levels in NSCLC patients show a stage-dependent pattern: in the early stages (I-II), differences in the level of HSP70 between patients with NSCLC and healthy individuals are statistically insignificant [29]. However, in the later stages (III-IV), there is a significant increase in the level of HSP70 compared with the control group [29]. The quantitative data are presented in Table 3. In the later stages of the disease, there is also a decrease in the number of CD4+ T helper cells and an increase in the number of CD3/CD94+ NK cells [29]. The prognostic significance of this immunophenotypic shift in the context of host immune competence is discussed in Section 4.3.

Despite the intuitive correlation between the level of HSP70 expression and the size of the primary tumor, no statistically significant relationship was found between these two characteristics [10,40]. However, eHSP70 release reflects an invasive migratory cellular phenotype rather than static tumor bulk: eHSP70 is actively secreted by cells undergoing epithelial–mesenchymal transition (EMT) as demonstrated in hepatocarcinoma cell lines (in vitro models) [43] and promotes extracellular matrix in vitro remodeling through MMP2 activation [44], processes that are mechanistically linked to metastatic dissemination rather than to primary tumor growth per se. Nevertheless, this marker effectively divides patients into groups with and without lymphogenous metastasis. In addition, eHSP70 levels are significantly higher in patients with early recurrence after surgery (see Table 3) [10] (clinical study, *n* = 178 NSCLC patients). At the same time, tumors with a negative PD-L1 status are characterized by significantly higher levels of eHSP70 compared to tumors with a positive status, representing a potential predictor of the response to immunotherapy, but additional investigation is required [10]. An operational approach worth pursuing in future prospective studies would be to calculate an eHSP70/GTV ratio derived from simultaneous serum lipHSP70 ELISA measurements and volumetric CT-based tumor segmentation [40], or alternatively to normalize eHSP70 to metabolic tumor volume (MTV) obtained from 18F-FDG PET-CT imaging [45]. Such a normalized index would formally dissociate the contribution of tumor mass from that of the invasive phenotype to the circulating eHSP70 signal, and could clarify whether eHSP70 retains independent prognostic value beyond what is already captured by standard volumetric staging parameters.

Unlike HSP70, which does not demonstrate significant efficacy in the early stages of lung cancer, serum HSP27 shows activity at all stages of disease [41]. The accuracy of diagnosis using HSP27 varies significantly depending on the commercial test system used [46]. Independent studies confirm a correlation between serum HSP27 and intratumoral expression, indicating that circulating HSP27 reflects a chaperone state characteristic of tumor cells [47].

In NSCLC, plasma HSP90α levels increase with disease progression, enabling the differentiation of patients with advanced disease from those with a favorable response to therapy [42]. High plasma HSP90α levels are associated with low sensitivity to first-line chemotherapy and reduced patient survival [42]. Quantitative data are presented in Table 3. Pre-treatment plasma HSP90α level has been shown to be an independent predictor of decreased survival in NSCLC patients receiving PD-1/PD-L1 inhibitors in combination with chemotherapy [48].

**Table 3:** Summary of studies reporting circulating eHSP levels in lung cancer patients.

eHSP	Cancer Type	Sample Type	Cancer Level	Control Level	Source
HSP70 (vesicular + free)	NSCLC all stages	Plasma	Median 125.4 ng/mL	16.4 ng/mL	[10]
HSP70 (vesicular + free)	NSCLC + lung mets	Serum/plasma	Mean 332.2 ± 37.9 ng/mL	35.1 ± 4.0 ng/mL	[33]
HSP70	NSCLC I–IV	Plasma	↑ stages III-IV only	Not elevated	[29]
HSP70	SCLC	Plasma	Mean 6.91 ng/mL	2.47 ng/mL	[49]
HSP27	NSCLC I–IV	Serum	↑ both early and advanced	Lower	[41,46]
HSP27 mRNA	NSCLC	Serum	Cutoff 25.4	Lower	[47]
HSP90α	NSCLC/LC	Plasma	Advanced > early	Lower	[42]
eHSP90α	SCLC	Plasma	High = poor chemo response	–	[50]
HSP90α	Advanced NSCLC + immunotherapy	Plasma	High = shorter PFS/OS	–	[48]
HSP90AA1	Pan-cancer	Plasma	Median 157.8 ng/mL	<69.19 ng/mL	[40]

NSCLC, non-small cell lung cancer; SCLC, small cell lung cancer; ↑, increased; mets, metastases; PFS, progression-free survival; OS, overall survival; LC, lung cancer.

#### 3.1.4. Colorectal Cancer: Stage-Independent Prognostic Stratification by Circulating HSP70 and Mortalin

Colorectal cancer (CRC) is one of the few malignancies in which the original eHSP70 observations were subsequently confirmed in an independent patient cohort [13,51]—a level of external validation that remains uncommon in the broader eHSP literature.

In CRC, serum eHSP70 levels generally do not exceed those observed in healthy individuals. This contrasts with other malignancies, such as non-small cell lung cancer (NSCLC) and glioblastoma (GBM), where elevated HSP70 concentrations are frequently detected. Importantly, serum eHSP70 levels in Stage I–II and Stage III CRC patients are in fact lower than the healthy control level, with no statistically significant difference between these groups [51] (see Table 4). A significant elevation above the control baseline is observed exclusively in Stage IV [51]. This pattern indicates that early-stage CRC cannot be reliably differentiated from healthy individuals on the basis of serum eHSP70 alone. A progressive intra-group increase across stages is nonetheless evident, and a prognostic threshold of >1.65 ng/mL reliably stratifies patients in a clinical study by survival risk regardless of stage [13].

Notably, the prognostic value of HSP70 is most evident specifically in patients without distant metastases. This observation is clinically significant, as these patients are conventionally classified as having relatively favorable prognosis by TNM staging: serum eHSP70 thus identifies a high-risk subgroup not captured by standard staging [13,51]. This approach addresses a clinical gap that CEA and CA19-9 are insufficient to resolve [13].

Mechanistically, the inflammation caused by oncological processes enhances HSF1-dependent transcription of HSP70. Furthermore, inflammatory cytokines stimulate eHSP70 vesicular release, creating a positive feedback loop that amplifies circulating eHSP70 levels [51].

Serum mortalin, also known as GRP75/HSPA9, is a mitochondrial HSP family member whose presence in serum is statistically independent of eHSP70 levels [52]. The two markers reflect distinct aspects of tumor biology. Combining mortalin and eHSP70 data provides a more accurate prediction than either marker alone or the standard clinical model that includes stages of disease, number of lymph nodes affected, CEA, CA19-9, and perioperative therapy information [52,53].

Elevated intratumoral HSP70 and HSP27 are independently associated with adverse survival in CRC [54], and elevated circulating eHSP70 likewise predicts poor outcomes across stages [13,51]. This prognostic impact of intratumoral and circulating HSP70 should not be conflated with the distinct prognostic significance of membrane-associated HSP70 in CRC, where its surface expression is associated with improved overall survival through enhanced NK cell-mediated immune recognition [55]. This distinction between protein pools may contribute to more effective treatment of patients with stages I-II of cancer who lack other markers to determine the need for adjuvant chemotherapy [54].

**Table 4:** Summary of studies on circulating and tissue eHSPs in colorectal cancer.

eHSP	Sample Type	Cancer Level	Control Level	Source
HSP70 (soluble)	Serum	>1.65 ng/mL	Lower	[13]
HSP70 (soluble)	Serum	Stage I–II: 1.79 III: 2.23 IV: 3.21 ng/mL	2.55 ng/mL (NS vs. CRC overall)	[51]
Mortalin (GRP75) + HSP70	Serum	Mortalin 10–214 ng/mL; independent of Hsp70	–	[52,53]
HSP70, HSP27	Tissue	High HSP70 = worse OS (*p* < 0.001)	–	[54]
HSPA1A, HSPA1B, HSPA1L	Tissue	High expression = worse OS	–	[56]

NS, not significant; OS, overall survival.

#### 3.1.5. Prostate Cancer: Multiple HSP Family Members as Complementary Markers of Tumor Aggressiveness

Among the malignancies discussed, prostate cancer (PCa) warrants particular attention for two closely related reasons. First, plasma HSP70 has emerged as one of the earliest biomarkers investigated in the context of liquid biopsy for PCa. Second, the clinical interpretation of these findings is complicated by the long-established use of prostate-specific antigen (PSA) as the standard biomarker for disease detection and monitoring.

In localized PCa, plasma HSP70 levels are elevated above those in healthy individuals [14]. However, in some patients with PSA levels within the normal range, plasma HSP70 levels also exceed the diagnostic threshold [14]. Despite this, a head-to-head comparison does not demonstrate higher diagnostic efficacy of plasma HSP70 compared to PSA [14].

In metastatic castration-resistant prostate cancer (mCRPC), the vesicular eHSP70 fraction is significantly higher than in localized disease [56]. This is correlated with the increased density of mHSP70 on the surface of androgen-independent tumor cells [56]. Magnetic beads targeting mHSP70 show significantly higher efficiency than the EPCAM-based method approved by the US Food and Drug Administration (FDA) [56]. Quantitative data are presented in Table 5.

In a tissue microarray study of 553 conservatively managed PCa patients, intratumoral HSP27 expression at diagnosis independently predicted poor clinical outcome regardless of ETS-gene rearrangement status [57]. Higher HSP27 staining was predominantly observed in tumors lacking ERG rearrangements [57], though this distribution did not carry independent prognostic significance. Intratumoral HSP60 remains an independent predictor of biochemical recurrence, even when other factors such as the Gleason score and extraprostatic spread are taken into account [58]. Complementing these tissue findings, serum HSP27 increases in proportion to Gleason score, but does not correlate with the level of PSA [59]. This independence from PSA renders serum HSP27 particularly informative in cases where PSA levels are ineffective, for example, in castration-resistant cancer, low-secreting tumors, and during androgen deprivation [59].

Plasma HSP90α levels are significantly elevated in PCa compared to benign prostatic disease and correlate with the regional lymph node status (N) and distant metastases (M), as well as the overall clinical stage [60]. This suggests that HSP90α level reflects the activity of tumor cell secretion rather than a nonspecific systemic background [60].

**Table 5:** Summary of studies on eHSP levels and expression in prostate cancer.

eHSP	Sample Type	Cancer Level	Control Level	Source
HSP70 (soluble)	Plasma	Localized: median 0.8 ng/mL	0.5 ng/mL	[14]
HSP70	Blood (vesicular + free)	Highly elevated (*p* < 0.0001)	–	[56]
HSP27, HSP60, HSP70	Tissue	HSP27 + HSP60 predict BCR; HSP70 does not	–	[58]
HSP27	Serum	Gleason 6: 6.6 → Gleason 8–10: 16.2 ng/mL	2.7 ng/mL	[59]
HSP27	Tissue	High = worse CSS and OS	–	[57]
HSP90α	Plasma	102.8 ± 89.8 ng/mL	62.6 ± 34.8 ng/mL	[60]

BCR, biochemical recurrence; CSS, cancer-specific survival; OS, overall survival; →, activates/leads to.

### 3.2. Hematological Malignancies

In hematological malignancies, tumor cells either circulate in the bloodstream or are closely associated with the hematopoietic system, which in principle facilitates detection of tumor-derived proteins in the circulation. However, the concurrent presence of reactive non-malignant populations—lymphocytes, myeloid progenitors, and stromal-derived cells—that also secrete HSPs in response to tumor-associated inflammation represents a major interpretive challenge specific to this disease category.

#### 3.2.1. Leukemias: Entity-Specific eHSP70 Profiles Across Acute and Chronic Subtypes

In 75% of acute myeloid leukemia (AML) cases, mHSP70 is detected on the surface of the blasts in bone marrow aspirates. In normal bone marrow, including hematopoietic precursors, and in the bone marrow of patients in complete remission, mHSP70 is not detected [61]. Consequently, blast cells carrying mHSP70 not only actively secrete vesicular eHSP70, but also become a target for NK cells—analogously to the mechanisms described for NSCLC and GBM (Section 3.1.1 and Section 3.1.3).

Plasma HSP70 levels vary significantly by disease entity: in acute myeloid leukemia and acute lymphoblastic leukemia (ALL), plasma HSP70 significantly exceeds that of healthy donors and patients with myelodysplastic syndrome (MDS). Notably, the median plasma HSP70 in ALL is higher than in AML [62]. Detailed data are presented in Table 6.

In MDS, plasma HSP70 does not significantly differ from healthy controls, consistent with the absence of mHSP70 surface expression and active vesicular secretion at this pre-malignant stage. The transition from MDS to acute myeloid leukemia is accompanied not only by an increase in the number of blast cells, but also by changes in proteostasis and chaperone expression [62]. These changes can be more accurately determined using plasma HSP70 than with conventional blast counting [62].

In chronic myeloid leukemia (CML), plasma eHSP70 levels are elevated above healthy donor values across all stages of the disease. However, no significant differences were found between the chronic and blast phases [63]. During the chronic phase, HSP70 levels correlate with faster disease progression and decreased patient survival [63].

#### 3.2.2. Lymphomas: eHSP as a Marker of the Stage and Response to Treatment in Conditions of Ambiguity of the Cellular Source

The interpretation of circulating HSPs in the context of lymphoma is complicated due to greater biological ambiguity than in leukemia. In classical Hodgkin’s lymphoma, neoplastic B cells are surrounded by a rich inflammatory microenvironment that includes reactive T cells, macrophages, eosinophils, and plasma cells, all of which can serve as HSP sources. Unlike AML, where mHSP70 permits direct identification of malignant blasts, lymphomas lack an equivalent cell-surface discriminator, rendering tumor-specific HSP attribution substantially more challenging.

An increased expression of the HSP90 is observed in HRS cells characteristic of classical Hodgkin’s lymphoma, where it plays a key role in ensuring the stability of oncogenic proteins, which are essential for the survival, growth, and evasion of tumor cells from the immune system [64]. The dependence of key oncogenic pathways on HSP90 explains the effectiveness of HSP90 inhibitors in various subtypes of lymphomas [64]. HSP90 expressed by circulating lymphocytes in lymphoma patients mainly originates from reactive non-clonal B lymphocytes, rather than from malignant clones [65].

In diffuse large-cell B-cell lymphoma (DLBCL), serum HSP70 and HSP90 levels correlate with Ann Arbor stage and decrease during R-CHOP treatment [66]. However, in patients who have not responded to treatment, serum HSP70 levels remain elevated after completion of R-CHOP therapy [66], indicating residual viable disease.

Co-detection of HSPs with tumor-specific surface markers on extracellular vesicles offers a strategy to resolve the cell-of-origin ambiguity. A significant increase in the number of circulating microvesicles with tumor-specific subpopulations has been documented in Hodgkin’s lymphoma, multiple myeloma, and acute myeloid leukemia [67]. When HSP70 and HSP90 are detected in vesicles together with markers such as CD19, CD30, or clonal light chains, tumor-specific EV subpopulations can be isolated [67,68].

#### 3.2.3. Multiple Myeloma: Compelling Mechanistic Rationale and Critical Translational Gap

Multiple myeloma (MM) presents a compelling but clinically under-explored context for eHSP-based liquid biopsy, with a strong biological rationale that has not yet been matched by systematic clinical evidence.

Unlike most solid tumors where HSP induction is driven primarily by hypoxia and necrosis, in MM the constitutive immunoglobulin secretory activity of plasma cells becomes a pathological burden sustaining constant endoplasmic reticulum stress and HSF1 activation [69]. Consequently, elevated levels of HSP70 and HSP90 are uniformly expressed across myeloma cells irrespective of proliferative status, whereas normal plasma cells with comparable immunoglobulin output do not exhibit this dependence. Malignant cells additionally rely on HSP90 to stabilize dysregulated oncogenic kinases and transcription factors [69].

Blocking HSP70 sensitizes MM cells to apoptosis in vitro [70]. Notably, HSP90 inhibition activates compensatory HSP70 upregulation via HSF1 derepression, which partially limits the anti-myeloma efficacy of HSP90 inhibitors [69]. Myeloma cell interactions with bone marrow stromal cells promote HSP70 expression and its vesicular secretion [71].

In HSP90 inhibitor trials in MM, intracellular HSP70 induction in peripheral blood mononuclear cells (PBMCs) served as a pharmacodynamic marker of target engagement, with HSP70 elevation confirming adequate drug exposure [72]. The combination of HSP90 inhibitor with bortezomib resulted in a specific increase in intracellular HSP70B (HSPH1) in MM cells and PBMCs, offering a biomarker to distinguish combination from monotherapy effects [73].

No systematic longitudinal eHSP70 monitoring data exist for MM patients from diagnosis through relapse—a research gap rather than a biological limitation, given the routine availability of serial plasma samples in this disease. Available quantitative data on HSP expression and circulating markers in MM and lymphoma are summarized in Table 7. Existing liquid biomarkers (e.g., serum M protein, free light chains) are uninformative in non-secretory forms of MM; minimal residual disease assessment requires bone marrow biopsy. Exosomal eHSP70, as a property of malignant rather than secretory plasma cell function, may in principle detect viable myeloma cells regardless of M-protein secretion status—a hypothesis warranting prospective clinical evaluation.

### 3.3. Are There Cancer-Specific eHSP Signatures?

Accumulating evidence supports the existence of cancer-specific extracellular heat shock protein (eHSP) signatures. The concept posits that extracellular chaperone profiles are shaped by tumor-specific biological contexts—including histological origin, genetic alterations, biological behavior, and therapeutic response [27,73]. The data are represented in Table 8.

These “signatures” are more than just the presence of a single protein. A diagnostically informative eHSP profile encompasses specific HSP family members implicated (e.g., eHSP70 versus eHSP90α); their compartmentalization (freely circulating versus vesicle-associated or membrane-bound); and their associated co-chaperone and client protein repertoires, which reflect the oncogenic signaling dependences of a given tumor [33,75].

Characterization of these molecular profiles may improve patient stratification and inform clinical decision-making by providing biological information not captured by conventional markers [76].

Additionally, cancer-specific eHSP signatures may serve as valuable tools for treatment monitoring. Dynamic changes in circulating eHSP levels (particularly the decreases observed during effective anticancer treatment) may provide means of monitoring tumor regression and evaluating therapeutic response, including response to radiotherapy [33,77].

**Table 8:** eHSP signatures in accordance with cancer type and their clinical significance.

eHSP Signature	Cancer Type/Context	Clinical Significance	Source
eHSP90α	Lung Adenocarcinoma (LUAD) with EGFR L858R mutation	High levels linked to shorter survival; better prognostic value than in 19DEL mutation.	[76]
eHSP90	Breast Cancer Stem Cells (BCSC)	Acts as a functional marker; targeting it reduces tumor growth.	[78]
Vesicular Hsp70	Glioblastoma, NSCLC	Elevated levels in patient blood serve as a tumor-specific biomarker for diagnosis and monitoring therapy.	[33]
Multi-HSP Complexes	Glioblastoma	Invasive cells release complexes of HSPs with specific inflammatory proteins (e.g., CHI3L1), forming unique “biomarker signatures”.	[75]
eHSP70 (in plasma)	Breast Cancer	Diagnostic Marker: Elevated levels in plasma samples compared to healthy controls. This suggests potential for early detection or monitoring.	[77]
mHSP70 (membrane-bound)	Gastric and Colon Carcinomas	Good Prognosis: Expression of membrane-bound Hsp70 correlates with improved overall survival. This may be due to enhanced immune recognition and elimination of these tumor cells.	[55]
mHsp70	Squamous Cell Carcinomas (e.g., Head & Neck, Esophageal)	Poor Prognosis: In contrast to gastric/colon cancers, mHsp70 expression is associated with a negative outcome. The reasons are unclear but may relate to different metastatic routes or immune environments.	[55]
eHSP70 and eHSP90	Melanoma	Progression Marker: Presence in plasma correlates with disease progression and metastasis, making it a marker for aggressive disease.	[77]
eHSP70 (in urine)	Ovarian Cancer	Diagnostic Marker: Elevated exosomal HSP70 levels in urine can distinguish cancer patients from healthy individuals, offering a non-invasive testing route.	[77]
HSP110, 90, 70, 60 families	Pan-Cancer Overview (33 types)	Tumor-Specific Roles: Overall, expression is linked to poor prognosis in respiratory, digestive, urinary, and reproductive system tumors, but to good prognosis in cholangiocarcinoma and pheochromocytoma/paraganglioma	[79]

Increasing evidence further indicates that extracellular HSPs are closely associated with cancer stem cell maintenance, tumor progression, and metastatic dissemination [80,81,82,83,84], rendering them attractive candidates for targeted intervention.

In summary, cancer-specific eHSP signatures represent a biologically grounded and clinically promising model, but require further systematic standardization and prospective validation across platforms and patient cohorts.

### 3.4. Physiological vs. Pathological Levels of eHSPs

#### 3.4.1. Altered eHSP Levels in Cancer

Levels of eHSP are significantly altered in cancer patients compared to healthy individuals. This alteration is not limited to a single cancer type but is a broad phenomenon observed across many malignancies, making eHSPs promising candidates for pan-cancer biomarkers.

Quantitative data for two circulating eHSPs—eHSP90α and eHSP70—across major malignancies are presented in Table 9. While eHSP90α consistently shows elevated levels in cancer patients compared to healthy controls, with the degree of elevation varying by cancer type, eHSP70 data show more variability, largely attributable to differences in the detection method (e.g., measuring free-floating vs. exosomal-bound eHSP70).

The degree of eHSP elevation often correlates with disease severity. For instance, serum HSP70 levels in lung cancer patients are significantly higher in stage IV disease (561.3 ng/mL for adenocarcinoma) than in stage III (260.3 ng/mL). This suggests that higher eHSP levels can indicate more advanced or aggressive disease. A crucial advancement is the ability to measure eHSPs specifically on tumor-derived exosomes. Research shows that exosomal HSP70 levels, rather than free-floating eHSP70 in the blood, correlate better with the HSP70 content within tumor biopsies and are more sensitive predictors of metastasis and treatment response. This represents a more refined and cancer-specific biomarker [24,33].

##### Early-Stage Disease

The detection of eHSPs in early-stage disease is particularly significant because it offers a potential window for intervention before cancer has spread. While eHSP levels generally rise with disease progression, substantial evidence confirms they are already altered in early-stage cancers (Stage I-II), making them valuable targets for developing early diagnostic tools [39].

The large-scale pan-cancer study found that plasma HSP90α levels are already elevated in early-stage cancers (Stage I-II), with diagnostic performance nearly identical to that seen in all stages combined. This is a powerful finding, suggesting that the physiological changes driving eHSP90α secretion occur very early in tumorigenesis [39].

The data on HSP70 initially seem contradictory but are resolved by understanding how the protein is measured. One study found that free circulating HSP70 levels were significantly decreased in early-stage lung cancer patients compared to healthy controls. This decrease, when combined with elevated traditional markers (CEA, CA19-9), achieved very high diagnostic accuracy (AUC 0.945). By contrast, a different study using the HSP70-exo ELISA, which specifically detects eHSP70 attached to tumor-derived microvesicles, found that these levels were already elevated in early-stage NSCLC compared to healthy individuals. This is a critical distinction: measuring vesicle-bound eHSP70 appears to be a more direct reflection of active tumor cell secretion, while free HSP70 levels may be influenced by other factors [10,87].

For non-melanoma skin cancers, the diagnostic approach focused on the body’s immune response to the tumor. A panel of autoantibodies (including anti-HSP70) was able to distinguish early-stage patients from healthy controls with excellent specificity (91.2%). This suggests that for some cancers, measuring the host’s humoral response to tumor antigens may be as valuable as measuring the antigens themselves [88].

##### Locally Advanced Cancer

In locally advanced cancer (typically Stage III, non-metastatic but invasive), eHSP levels are not just elevated; they are dynamic biomarkers that reflect active tumor biology and can predict how a patient will respond to treatment. Unlike in early-stage disease, where eHSPs help with diagnosis, in locally advanced disease their primary clinical value lies in monitoring therapy and predicting outcomes.

During treatment in locally advanced cancer eHSP70 has a “Dual Role”. This is the most critical concept. During radiotherapy, dying tumor cells release eHSP70, which acts as a “danger signal” to alert the immune system. Therefore, higher post-treatment eHSP70 levels are actually a good sign, indicating that the treatment is killing cancer cells in a way that stimulates an anti-tumor immune response. The ideal scenario is a significant drop in overall eHSP70 (showing reduced tumor mass) combined with a post-treatment elevation (showing immune activation) [33,89].

While eHSP70 is great for monitoring, eHSP90α is more directly involved in the biology of locally advanced cancer and acts as an invasive factor. It is actively secreted by tumor cells to remodel the surrounding environment, breaking down barriers and allowing the cancer to invade locally. This makes eHSP90α a potential therapeutic target to stop locally advanced tumors from becoming metastatic [90].

#### 3.4.2. eHSPs in Severe and Advanced Cancer

In severe and advanced cancer (stages III-IV), eHSPs play dual, complementary roles. They are not merely elevated, they are actively driving the aggressive features that define this stage: relentless invasion, metastasis, and therapy resistance.

##### Metastatic Disease

In thoracic cancers, levels of eHSP70 (specifically the vesicle-bound form) are a direct indicator of disease severity: eHSP70 levels are low in early-stage disease, higher in locally advanced cancer, and highest in metastatic disease. Patients with lymph node metastases show significantly elevated eHSP70 levels prior to surgery. Moreover, eHSP70 actively helps cancer cells survive standard therapies, making it a key player in treatment failure. Furthermore, high baseline levels predict early relapse even after complete tumor resection [10,73,87].

##### Therapy-Resistant Tumors

In therapy-resistant tumors, eHSPs help cancer cells survive treatment through several key mechanisms: ejecting drugs via extracellular vesicles, blocking cell death pathways, and hijacking the stress response to evade the immune system.

Cancer cells adapt to HSP90 inhibitors by mutating the HSP90AA1 gene and overexpressing eHSP90α. This is a key mechanism of acquired resistance [91].

Stressed tumor cells, especially those undergoing epithelial–mesenchymal transition (EMT), increase their production and release of extracellular vehicles (EVs) loaded with pro-resistance cargo, including various eHSPs. This phenomenon is known as Resistance-Associated Secretory Phenotype (RASP). RASP not only helps the cell itself survive but also allows it to “share” resistance with neighboring, therapy-sensitive cancer cells through EV transfer, as seen with DNAJB8 (HSP40 family) in colon cancer. Through RASP, therapy-resistant tumor cells can also release eHSP-rich oncosomes that carry oncogenic factors to recipient cells, promoting resistance against hypoxia, radiation, drugs, and immune attack [80,91,92].

The p53 protein is a key transcription factor and tumor suppressor, and cancer cells often evolve ways to inactivate it, often using eHSPs for this. For example, in paclitaxel-resistant breast cancer, the eHSP gp96 (an ER-resident HSP90) promotes the degradation of p53, effectively removing a key barrier to cell survival. Packaged into small EVs and transferred from resistant to sensitive cells, DNAJB8 stabilizes p53, which upregulates the drug efflux pump MDR1 to drive Oxaliplatin resistance [92,93].

Meanwhile, eHSP70 has a dual role that becomes critical in therapy resistance. Acute release eHSP70 at its low chronic levels (e.g., from dying tumor cells during therapy) activates the immune system and enhances treatment response. Chronic high levels of eHSP70 (e.g., in advanced, resistant tumors) induce immune tolerance and promote treatment failure. This concentration-dependent duality means that in therapy-resistant tumors, persistently elevated eHSP70 levels actively suppress the anti-tumor immune response rather than activating it [26,94].

Thus, eHSP70 and eHSP90 contribute to therapy resistance through distinct but complementary mechanisms: eHSP90 primarily drives resistance through RASP, drug ejection via oncosomes, and stabilization of mutated oncoproteins; eHSP70 contributes through membrane stabilization and immune modulation, becoming immunosuppressive when chronically elevated in resistant tumors. The effects of eHSP70 and eHSP90 are mediated through specific receptor interactions (Table 10).

## 4. Biological Roles of eHSPs in Cancer

Beyond their biomarker role established in Section 3.1 and Section 3.2, extracellular HSPs actively shape the tumor microenvironment—the same molecules can drive tumor growth and immune evasion while activating anti-tumor immune responses in other cellular contexts.

### 4.1. Tumor-Promoting Effects of eHSPs

In healthy tissues, eHSP90α supports regeneration through extracellular matrix remodeling and cell motility activation, including angiogenesis [95]. In malignant cells, these processes become constitutive and unregulated, driving pathological matrix restructuring, invasion, and immune evasion [95]. Vesicular eHSP70 exhibits context-dependent duality, promoting tumor cell survival while simultaneously suppressing anti-tumor T cell responses via myeloid-derived suppressor cells [96]. The differences between the pro-tumor and anti-tumor functions of eHSPs depend on the protein structure, the type of host cell, and the inflammatory environment in tumor tissue. This context-dependence of pro- and anti-tumor eHSP functions is discussed in Section 4.3.

#### 4.1.1. Tumor Growth and Survival

Extracellular eHSP70 has been proposed to activate NF-κB and PI3K/AKT signaling in tumor cells through TLR4 and RAGE receptors, thereby promoting proliferation and suppressing apoptosis [73]. If operative, this mechanism would not be fully addressed by approaches targeting the intracellular HSP70 pool alone, and may function independently of intracellular HSP70’s anti-apoptotic activity [73]. Consistently, elevated circulating eHSP70 in NSCLC, CML, and ALL is associated with poorer survival (Section 3.1.3 and Section 3.2.1).

A similar feedback loop operates through eHSP90α via LRP1: increased resistance to apoptosis under hypoxic conditions is a factor that stimulates protein secretion and accelerates the cell cycle due to activation of AKT, ERK, and NF-κB [97,98]. Elevated plasma eHSP90α in late-stage lung cancer (Section 3.1.3) and its correlation with metastatic stage in prostate cancer (Section 3.1.5) are consistent with this LRP1-mediated survival program.

#### 4.1.2. Angiogenesis and Invasion

The invasion is based on the interaction between eHSP90α and matrix metalloproteinase 2 (MMP2). Extracellular eHSP90α activates this extracellular protease, degrading basement membrane components, including collagen IV, fibronectin and laminin [97,98], creating conditions for invasion and neoangiogenesis. Activated MMP2 can release vascular endothelial growth factor (VEGF) from the matrix, stimulating the migration of endothelial cells [97]. This process is regulated by a molecular switch: co-chaperone AHA1 competes with TIMP2 for the binding site on the middle domain of eHSP90α. The displacement of TIMP2 reactivates MMP2, driving increased invasion [98]. Inhibition of eHSP90α using non-cell-penetrating antibodies prevents MMP2 and MMP9 activation, reducing lung metastases in preclinical experimental models of breast cancer and suppressing invasion in glioblastoma and lymph node metastasis in melanoma [97,98].

In various tumor types, eHSP90α promotes EMT, a process that enhances the invasive and metastatic potential of cancer cells, through context-dependent mechanisms. In breast cancer, eHSP90α cooperates with clusterin through LRP1 signaling to downregulate E-cadherin and upregulate mesenchymal markers (N-cadherin, Slug, Snail, ZEB1) [98]. In prostate cancer, eHSP90α suppresses E-cadherin through EZH2 [97]. In these mechanisms, LRP1 plays a central role as a key mediator of extracellular HSP signaling, making it a potential therapeutic target. eHSP70 acts as an essential cofactor of the invasive machinery: its depletion from the conditioned medium specifically attenuates eHSP90α-mediated activation of matrix metalloproteinase-2 (MMP-2) [98].

The role of eHSP90α in angiogenesis is not limited to VEGF release. Under hypoxic conditions, melanoma cells produce small extracellular vesicles containing HSP90 and IKKα/β in an in vitro melanoma model [84], activating NF-κB/CXCL1 in tumor-associated fibroblasts to stimulate angiogenesis via a paracrine pathway [97]. With regard to lymphangiogenesis, as detailed in Section 3.1.2, eHSP90α acts via LRP1 on lymphatic endothelial cells to activate AKT and increase CXCL8 levels [25]. The parallelism of these two mechanisms, acting through LRP1/AKT, suggests a coordinated pro-invasive program spanning both vascular compartments.

#### 4.1.3. Immune Suppression

The mechanism by which vesicular eHSP70 suppresses the immune response is associated with TLR/NF-κB receptors. In dendritic cells, TLR4 signaling activated by eHSP70 promotes adaptive immunity. In myeloid-derived suppressor cells (MDSCs), TLR2 signaling activated by exosomal HSP72 has the opposite effect.

Exosomal HSP72 interacts with TLR2 on MDSCs, initiating the production of interleukin-6 through the MyD88 adapter, which leads to phosphorylation of STAT3 [96]. Activated STAT3 drives immunosuppression via production of arginase-1, iNOS, and TGF-β [96]. Blocking exosome release using amiloride significantly reduces MDSC activation and enhances the anti-tumor effect of cyclophosphamide in vivo [96].

A complementary immunosuppressive pathway operates through tumor-associated macrophages (TAMs). Chemotherapy triggers the release of eHSP70 from tumor cells, stimulating TGF-β-driven M2 polarization, marked by elevated CD163, IL-10, and MMP2 [99,100] as demonstrated in vitro with clinical validation in 116 breast carcinoma tissue specimens [99], promoting resistance to chemotherapy and migration of tumor cells. Experimental blockade of eHSP70 release reverses M2 polarization and restores chemotherapy sensitivity in vitro [100].

The pro-tumor mechanisms described above are largely mediated by vesicle-associated eHSPs, underscoring the diagnostic relevance of the vesicular fraction over free-soluble eHSP70 for assessing tumor invasive and immunosuppressive potential [96,100]. The main mechanisms underlying these processes are presented in Table 11.

### 4.2. Anti-Tumor and Immunostimulatory Role of eHSPs

The pro-tumorigenic effects described in Section 4.1 represent only one pole of HSP biology. Under conditions of intact host immunity, the same molecules can activate innate and adaptive anti-tumor responses—a duality already evidenced in the clinical biomarker data from grade III gliomas, CML, and post-radiotherapy NSCLC [11,63].

#### 4.2.1. eHSPs as Danger-Associated Molecular Patterns and the Paradox of TLR

eHSPs function as DAMPs—endogenous molecules released from damaged or dying cells that signal tissue injury to the immune system through pattern-recognition receptors [73,101]. When HSP70 binds to Toll-like receptor 4 (TLR4) on the surface of dendritic cells and macrophages, NF-κB activation drives production of tumor necrosis factor-α (TNF-α), interleukin-1β (IL-1β) and interleukin-12 (IL-12) [73,101]. This contrasts with TLR2-dependent STAT3 activation, discussed in Section 4.1: both processes operate via TLR/MyD88, but produce opposite immune outcomes: Th1 activation through dendritic cells versus T cell suppression through MDSCs. The determining factor is the type of target cell, and not the ligand as such. In the context of biomarker research, this means that the level of circulating eHSP70 protein cannot serve as a reliable indicator of immune system activation without concurrent assessment of the myeloid compartment composition.

mHSP70 is selectively expressed on a broad range of tumor cell surfaces but is absent from normal tissues and from bone marrow of patients with leukemia in complete remission [28,61]. This phenomenon is associated with the interaction of HSP70 with glycosphingolipid Gb3 in cholesterol-enriched microdomains and its ability to bind to phosphatidylserine on the outer surface of tumor membranes [28,61].

#### 4.2.2. NK Cell Activation Through the mHsp70-TKD-CD94 Axis

The best-characterized immunostimulatory function of mHSP70 is specific NK cell activation via the CD94/NKG2C receptor. NK cells with the activating CD94/NKG2C heterodimer on their surface recognize membrane-bound HSP70 located on tumor cells. After that, they begin to destroy these cells, bypassing the classic MHC class I presentation mechanism [28,102,103]. The functional domain responsible for the activation of the immune system is located in the 14-amino acid C-terminal peptide TKD (aa450–463) [102,103]. Pre-stimulation of NK cells in patients using a combination of interleukin-2 and the TKD peptide significantly increases their ability to destroy HSP70-positive targets [103]. Molecular docking and knockout performed using the CRISPR/Cas9 system allowed us to determine that CD94 is a key partner for TKD binding. Blocking CD94 completely suppresses NK cell activation caused by TKD [104].

Ex vivo stimulation of autologous NK cells using TKD and IL-2 significantly increases their ability to destroy eHSP70-expressing cells in ten out of twelve patients with advanced tumors in a phase I clinical trial [105]. In a randomized phase II trial, patients with non-small cell lung cancer who received activated NK cells after radiotherapy demonstrated significant improvement in tumor control [106].

These clinical findings are mechanistically supported by the biomarker data from Section 3.1.1: in grade III gliomas, elevated vesicular eHSP70 associates with increased CD3−/CD56+/CD94+/CD69+ NK cell frequency and improved overall survival [11]. In contrast, glioblastoma’s collapsed CD4+ compartment renders the same signal immunosuppressive—a direct illustration of how TME immune competence determines the biological outcome of equivalent eHSP70 levels.

#### 4.2.3. Cross-Presentation and Priming of Adaptive Immunity

eHSPs bridge innate and adaptive immunity by chaperoning tumor-specific peptides into the MHC class I cross-presentation pathway of antigen-presenting cells [107]. eHSPs are not antigens per se; Binder and Srivastava demonstrated in mouse tumor models that the immunogenicity of gp96- or HSP70-peptide complexes depends on the characteristics of the peptide cargo of a particular tumor, and not on the HSP molecule itself [108]—a finding that underpins the rationale for personalized HSP-peptide vaccines.

Cross-presentation is mediated by CD91/LRP1 on dendritic cells and macrophages [107,108]. After internalization, HSP90 transports the antigen through the Sec61 translocon into the cytosol [107,109]; the proteasome then generates peptides loaded onto major histocompatibility complex class I, activating CD8+ cytotoxic T-lymphocytes [107,109]. HSP70 and HSP90 perform complementary roles: HSP90 mediates transmembrane antigen transport, while HSP70 ensures its further processing [109].

CD4+ T helper activation occurs in parallel through MHC class II, facilitated by co-receptors LOX-1 and SREC-1 [110]. Simultaneous blockade of these co-receptors with CD91 suppression abrogates HSP70-driven CD4+ T cell proliferation [110], and HSP90 additionally mediates cross-presentation via SREC-I [111]—explaining why multichaperone HSP vaccines elicit a more balanced adaptive immune response than single-antigen peptide formulations [110,111].

In a phase I GBM trial, autologous gp96-peptide complexes from resected tumors induced IFN-γ production upon re-administration to patients with recurrent disease [112].

#### 4.2.4. The CD91 Paradox as an Integrating Concept

CD91/LRP1 performs both pro-tumor and anti-tumor functions: it mediates immune activation through eHSP-peptide complex recognition on dendritic cells [107,108] and simultaneously drives tumor invasion through eHSP90α-AKT signaling [97]. In lymphatic endothelial cells, CD91/LRP1 stimulates lymphangiogenesis via eHSP90α [25]. In advanced cancer, monocyte CD91 expression decreases in the peripheral blood, reducing sensitivity to eHSP-derived immunostimulatory signals and impairing cross-presentation and NK cell activation [113]. This CD91 downregulation, accompanied by MDSC expansion and CD4+ T cell depletion, forms a complex immunological barrier that prevents eHSP-mediated anti-tumor responses. A high circulating eHSP70 level with intact immunity signals favorable prognosis, whereas the same level in an immunosuppressed host may indicate an unfavorable outcome.

### 4.3. Context-Dependent Duality

Depending on the concentration of eHSPs, the specific tumor microenvironment, the type of immune cells present, and their location (free vs. vesicle-bound), the exact same molecule can either stimulate a powerful anti-tumor immune response or promote tumor invasion (Table 12).

Free eHSPs might carry antigenic peptides to stimulate immunity or, if in high concentration, can induce immune tolerance. Exosome-bound eHSPs (mostly pro-tumor): this form is particularly dangerous. By packaging eHSPs inside exosomes, tumors can efficiently deliver their pro-metastatic and immunosuppressive cargo over long distances [117,118].

The key contextual determinants of this functional switch, such as cell death mode, eHSP location (membrane-bound vs. vesicular vs. free-soluble), local concentration, and receptor repertoire on target immune cells, are summarized in Table 12 and Table 13 and discussed in the subsections below.

#### 4.3.1. Concentration-Dependent Effects

**Figure 1:**
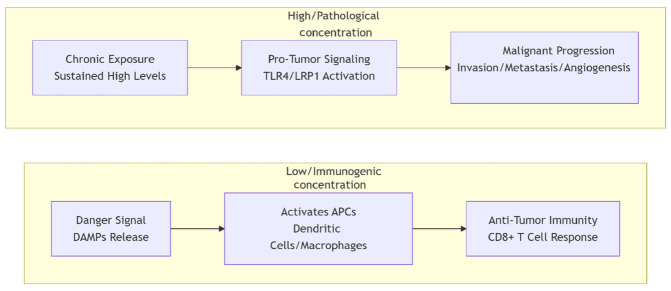
Concentration and the dual nature of eHSPs.

eHSP concentration is a primary determinant of functional outcome (Figure 1). Under physiological stress, eHSP90α promotes tissue repair through LRP1/CD91-mediated cell migration [119]. At transient low concentrations, eHSPs act as DAMPs, activating antigen-presenting cells and CD8+ T cells [114]. In contrast, the chronically elevated eHSP concentrations characteristic of advanced malignancies persistently activate TLR4 and LRP1, shifting the balance toward invasion, metastasis, and angiogenesis [98,114].

At levels found during normal physiological stress (physiological to moderate), eHSPs function to promote cell migration and tissue remodeling. This is a normal, controlled process for wound healing [100].

At lower, transient levels, eHSPs act as Danger-Associated Molecular Patterns (DAMPs). They bind to antigen-presenting cells (APCs), facilitating the cross-presentation of tumor antigens and activating CD8+ T cells to attack the cancer and have immunogenic properties [114]. In cancer, eHSPs levels are the critical switch. Chronically high levels of eHSPs (e.g., eHSP90α in pancreatic cancer, which can be 100 times higher than in healthy individuals) shift the balance. These high concentrations are already pathological and persistently activate receptors like TLR4 and LRP1 on cancer and endothelial cells, driving invasion, metastasis, and angiogenesis [98,114].

The sustained high concentration of eHSPs fundamentally alters cellular behavior. One of the earliest signs of malignant change is the breakdown of communication between normal cells. Research shows that high concentrations of eHSP70 disrupt gap-junction intercellular communication (GJIC) between microvascular cells. This action literally dismantles the structural integrity of normal cell barriers, a key step that allows tumors to invade surrounding tissue. The sheer quantity of eHSP90α in the tumor microenvironment (TME) does more than just signal; it actively remodels the physical landscape. eHSP90α forms complexes with co-chaperones to activate matrix metalloproteinases (MMPs), enzymes that chew through the extracellular matrix (ECM) to clear a path for metastatic cells [98,119].

#### 4.3.2. Receptor Usage (TLRs, Scavenger Receptors, CD91, LOX-1)

The receptors that eHSPs bind to, specifically TLRs, CD91/LRP1, SREC-1, and RAGE, are the molecular switches that determine whether an eHSP signal promotes anti-tumor immunity or drives pro-tumor malignancy (Table 13).

**Table 13:** Main eHSP receptors and their dual roles in cancer.

Receptor	Primary eHSP Ligands	Anti-Tumor/Immunostimulatory Role	Pro-Tumor/Pro-tumoral Role
TLR2/TLR4	eHSP70, eHSP90, eHSP60	Acts as a DAMP; activates NF-κB in APCs, leading to pro-inflammatory cytokine release and T cell activation [81].	On cancer cells, it activates AKT and ERK signaling, driving invasion (e.g., in glioblastoma) and epithelial–mesenchymal transition (EMT) [81,120].
CD91 (LRP1)	eHSP70, eHSP90, gp96	On APCs, mediates cross-presentation of HSP-chaperoned antigens, which is crucial for priming anti-tumor CD8+ T cell responses [81].	Currently, its primary described role is in immune activation; fewer direct pro-tumor functions on cancer cells have been identified [81].
SREC-1	eHSP70, eHSP90	Acts as a co-receptor with TLRs on APCs, essential for antigen uptake and cross-priming of T cells [81].	In cancer cells, this same pathway promotes survival, proliferation, and metastasis. In lung cancer, eHSP70-RAGE signaling enhances tumor aggressiveness [118].
RAGE	eHSP70, eHSP90, HMGB1	Activates NF-κB and ERK1/2, leading to pro-inflammatory cytokine production, which can contribute to immune cell recruitment [118].	Can also be immunosuppressive; its role in eHSP biology is less defined but context-dependent [81].
CD94/NKG2A	Membrane-bound eHSP70	Expressed on NK cells and CTLs; recognizes mHSP70 on stressed/tumor cells, triggering direct cytotoxicity [81].	On cancer cells, it activates AKT and ERK signaling, driving invasion (e.g., in glioblastoma) and epithelial–mesenchymal transition (EMT) [81,120].

The same eHSP can have opposite effects depending entirely on which receptor it binds to and on which cell type that receptor is expressed. The clearest example of this duality is CD91/LRP1. When eHSPs bind to CD91 on antigen-presenting cells (APCs), they activate a crucial pathway for cross-presentation, allowing the immune system to recognize and attack tumor antigens. However, when eHSP90 binds to the exact same receptor on cancer cells (e.g., in glioblastoma), it triggers signaling cascades (AKT, ERK) that promote cell motility, invasion, and metastasis [81,120].

Moreover, eHSP90α can directly bind to TLR4 on glioblastoma cells. This binding transactivates the Epidermal Growth Factor Receptor (EGFR), leading to calcium signaling and increased cell migration, bypassing the need for immune cells entirely. This demonstrates a direct, non-immune pro-tumor function for a classic immune receptor [121].

SREC-1 (Scavenger Receptor expressed by Endothelial Cells-1) appears to be more specialized. Its primary described role is on APCs, where it acts as a co-receptor for TLRs and is essential for the uptake of eHSP–antigen complexes and the subsequent cross-priming of T cells. Its direct role in cancer cell biology is less established [81].

RAGE is another pattern-recognition receptor. Studies confirm that eHSP70 acts as a direct agonist for RAGE, activating ERK1/2 and NF-κB in lung cancer cells. This leads to the expression of pro-inflammatory and pro-survival genes, directly contributing to a more aggressive tumor phenotype [118].

In summary, the specific receptor engaged thus functions as the primary determinant of eHSP signaling outcome. This complex network of receptors on both immune and cancer cells allows the same molecule to be both a guardian and a promoter of cancer progression [121].

#### 4.3.3. Tumor Type and Host Immune Status

The interaction between eHSPs and the host immune system is not a one-size-fits-all phenomenon but a dynamic interplay heavily influenced by tumor type and host immune status. The same eHSP can either activate an anti-tumor response or promote immune evasion, depending on the specific cancer and the immune cell populations present [23,122].

Different cancers create unique microenvironments that dictate how eHSPs behave. The clearest examples come from studies on pancreatic cancer, which is notoriously immunosuppressive. In Pancreatic Ductal Adenocarcinoma (PDAC), an aggressive tumor type, eHSP90α is a major driver of malignancy. Research shows that it binds to the CD91 receptor on macrophages, forcing them into a pro-tumor M2-like state. These M2 macrophages then suppress T cell activity and create a feedforward loop that produces even more eHSP90α, maintaining an immunosuppressive environment. The development of a humanized antibody (HH01) that blocks the eHSP90α–CD91 interaction has shown promise in in vitro and in vivo preclinical models, reducing M2 macrophages and reinvigorating T cell immunity [123].

Research on non-small cell lung cancer (NSCLC) highlights the dual nature of eHSP70. While eHSP70 is generally associated with aggressive tumors, clinical data shows that patients with higher post-radiotherapy eHSP70 levels actually had significantly better responses to treatment. This occurs because eHSP70 released from dying tumor cells acts as a “danger signal,” stimulating the immune system. Therefore, whether the eHSP is actively secreted by viable cells or passively released during cell death determines its immunological effect [80].

Furthermore, the host’s own immune competence and the expression of specific receptors on immune cells are equally critical. The receptor CD91 (also known as LRP1) is central to the function of eHSPs. On one hand, it is essential for antigen-presenting cells to take up eHSP-chaperoned peptides and cross-present them to T cells, which is crucial for initiating an anti-tumor response. Evidence indicates that in acute inflammatory states (like those found in cancer), a population of CD91-low monocytes expands. This downregulation of CD91 could represent a mechanism by which the host immune system becomes desensitized to eHSP “danger signals,” contributing to immune evasion [89,124].

The host immune status also determines the success of therapies targeting eHSPs. Unlike normal cells, many solid tumor cells (including lung, pancreatic, breast, and colorectal cancers) selectively express HSP70 on their cell surface (mHSP70). This makes mHSP70 a tumor-associated antigen and an accessible target for natural killer (NK) cells, which can recognize and kill these mHSP70-positive tumor cells. This is a direct example of how a patient’s innate immune status (the presence and activity of their NK cells) can be leveraged to target a tumor-type-specific eHSP signature [89].

## 5. Modulation of eHSP Levels by Interventions

### 5.1. Physical and Lifestyle Interventions

Physical exercise is widely recognized as a powerful non-pharmacological intervention that promotes health and aids in the prevention and management of numerous diseases. Owing to the broad spectrum of molecular, cellular, and systemic adaptations elicited by both acute exercise and chronic training, it has been described as a “polypill” [125]. Despite its undeniable benefits, it has only been considered in the adjuvant oncological treatment for a few years, mostly to prevent muscle loss and enhance oncological patients’ quality of life.

Both cancer and treatment are known to jeopardize functional capacity and cardiorespiratory fitness [126,127,128]. Different protocols of exercise have shown to work otherwise, by helping oncological patients in different stages. A 16-week protocol of physical training improved anthropometrical and functional parameters (strength, functional capacity and flexibility) in women during conventional treatment [129]. Similarly, exercise prehabilitation prior to and early after surgery also helped patients with oesophageal, gastric, head, neck and breast cancer to achieve physical fitness [130,131,132], therefore reassuring its power in oncological cotreatment.

As detailed throughout this manuscript, either tumoral growth or treatment are built upon changes in the immunological behavior. In this sense, in parallel to the effects on lean mass and quality of life, exercise has a time-dependent modulatory role on immunity [125]. A single bout of moderate-vigorous exercise temporarily enhances blood pressure and shear forces, leading to a profound mobilization of NK cells, CD8+ T cells and γδ T cells into the peripheral circulation [133]. Then, a transient decrease in blood lymphocyte counts follows about 1 or 2 h after the session, and returns to baseline levels within 24 h, characterizing the “post-exercise window” [133]. However, studies have shown that this transient lymphopenia does not mark immunosuppression, but a redistribution of circulating leukocytes to the tissues. Indeed, NK cell cytotoxic capacity against lymphoma and multiple myeloma cell lines increases by 60% one hour after exertion [134], characterizing a “hot tumor” with more immunological activity [125].

During the immediate response to an acute exercise bout, the body undergoes a broad physiological recalibration involving shifts in energy expenditure, ionic homeostasis, hormonal and cytokine dynamics [135], and heat production, collectively contributing to HSP expression and release [136]. Studies have shown that the leukocyte activity and HSP levels follow the same pattern of the immune system. Both acute sessions and short-term continuous incremental treadmill training increase the HSP70 expression in human PBMC in 24 h, further returning to baseline [137,138]. Mesenteric lymphocyte HSP70 expression reinforces this statement, reaching the peak four hours after a single bout of exercise [138]. Not surprisingly, both eHSP70 and eHSP27 release (passive or active) are enhanced by an exercise session, and then tend to go back to baseline in the recovery period (2 to 7 days after marathon) [139]. Together, these studies support the temporal dynamics of immunity and HSP behavior in response to exercise.

In addition to the temporal dynamics, exercise also has a dose-dependent modulatory role on immunity [140,141]. This field of study was pioneered by Doctor David Nieman and colleagues, who prospectively followed up sedentary, active subjects, runners, athletes and elite athletes for decades [140,141]. They observed that while a sedentary lifestyle offers no immune benefit, extenuating exercise/training may act otherwise by creating an “open window” for infections due to its immune-suppressive effects [140,141]. According to their studies, the optimal stimulus for immune function locates in the middle of the U-shaped curve, representing a moderate-intensity exercise that improves host defense by boosting innate and adaptive immunity [140,141].

Moderate-intensity exercise (40–70% VO_2_max for 10–60 min) and high-intensity interval training (80–100% VO_2_max for 1–4 min interspersed with 30–40% VO_2_max recovery for 1–3 min) enhance immune function across virtually all major leukocyte populations in health [141] and patients with stages I, II and III, non-metastatic and hormone-responsive breast cancer [142]. These adaptations include increased phagocytic capacity of neutrophils and monocytes, greater alternative polarization of macrophages, enhanced T cell proliferation, improved antibody production by B cells [141], and lower release of TNF-α, IL-6, and IL-10 [142]. In contrast, very strenuous and prolonged exercise (≥70% VO_2_max for ≥60 min or until exhaustion) does not elicit comparable immunological benefits [141]. However, in the oncological treatment, this U-shape relationship tends to get “delayed”, with more intensity being required to significantly affect immunity. According to a systematic review, vigorous-intensity exercise also benefits immunity, by promoting natural killer cell cytotoxicity, while light-to-moderate and moderate-intensity exercise results in no significant immunological improvements, particularly for patients undergoing active treatment [143].

Studies with animal models of different intensities of exercise training have reported workload-dependent changes in HSP expression and cellular function in leukocytes. While moderate to intense exercise enhances HSP70 expression in peripheral monocytes [144] and in mesenteric lymphocytes [138], and stimulates their phagocytosis [144] and proliferation [138], exhaustive exercise acts otherwise. The increase in workload-dependent HSP70 expression and release by mesenteric lymphocytes after exercise follows an increase in their proliferation and a suppression of NF-κB activation and nuclear localization [138]. Conversely, the lymphocytes from animals undergoing extenuating exercise had a higher IL-2-to-IL-10 ratio and did not express HSP70 proportionally [138]. The eHSP70 release by leukocytes enhances with exercise intensity, reaching huge levels on extenuating intensity, and may contribute to the increase in its plasmatic levels in healthy humans [139,145]. In support of this, studies show that the closer athletes work to 80% VO2 max, the higher their plasma eHSP70 levels [22,146,147].

As previously detailed in this manuscript, the roles of eHSP70 depend on its source, target and the way it was released, whether actively in microvesicles by stressed viable cells or passively by necrotic cells. While tumor-derived eHSP70 has pro-tumoral roles [18,19], the leukocyte-derived eHSP70 whose release is stimulated by exercise can elicit anti-tumoral activities [15,16,17]. Additionally, chronically high levels of eHSPs trigger a cycle of muscle loss and body-wide inflammation, sustained by their direct effect on muscle cells via the TLR-4-NF-κB pathway [118,148], and their inflammatory roles on peripheral leukocytes, creating a feedback loop that drives the cachexia syndrome [116]. Therefore, the workload-dependent effects of exercise on the eHSPs should be taken into account to reach a perfect intensity to “heat” tumors.

### 5.2. Cancer Therapies

The effect of cancer treatment on circulating eHSPs is multifaceted and of considerable clinical significance. Each therapeutic modality disrupts tumor cell proteostasis through a distinct mechanism, and each provokes a distinct eHSP response that can reflect, in real time, what is happening inside the tumor. Interpreting these responses requires distinguishing between two sources that are often conflated: the eHSP signal from dying tumor cells, which is largely free-soluble and immunostimulatory, and the signal from viable stressed-but-surviving cells, which is predominantly vesicle-associated and pro-tumorigenic. These two pools move in opposite directions after effective treatment, yet most assays capture their sum. The sections below examine how each major therapeutic modality perturbs eHSP levels, what those perturbations mean biologically, and what they may eventually mean clinically.

#### 5.2.1. Radiotherapy

The most longitudinally rigorous eHSP monitoring data derive from radiotherapy studies, where validated assay platforms and prospective cohort designs permit mechanistic interpretation of temporal response trajectories. The response trajectory, a substantial early fall followed by an incomplete return toward normal, has a straightforward biological interpretation: ionizing radiation kills a fraction of mHSP70-positive tumor cells acutely, reducing the vesicular HSP70 secretion that constitutes the dominant eHSP70 pool in advanced disease, while the residual elevation after treatment completion reflects both surviving viable tumor cells and the free-soluble HSP70 released from radiation-induced necrotic debris.

Before this fall-and-plateau picture became established, a seemingly contradictory observation created interpretive confusion that is worth examining directly because it reveals something important about the biology. In a prospective cohort study of NSCLC patients undergoing radiotherapy, those who achieved clinical response had paradoxically higher post-treatment serum HSP70 than non-responders, with patients above a cutoff of 4.35 ng/mL showing significantly better survival [80]. This apparent paradox resolves when assay sensitivity is taken into account: the R&D DuoSet used in that study preferentially detects the free-soluble fraction released by dying cells, whereas the compHSP70 ELISA predominantly captures the vesicular fraction from viable mHSP70-positive cells. In responders, effective RT generates more immunogenic tumor cell death and therefore more free-soluble HSP70—which yields high values by the DuoSet but contributes relatively little to the compHSP70 signal that tracks the viable vesicle-secreting tumor mass. In non-responders, fewer cells die and the radioresistant vesicle-secreting mHSP70-positive population persists largely intact, giving a higher compHSP70 but lower DuoSet reading, a demonstration of how assay selection determines the direction of the biomarker signal and why studies that aggregate DuoSet and compHSP70 data cannot be interpreted uniformly; studies aggregating DuoSet and compHSP70 data should not be interpreted interchangeably [80].

In breast cancer treated with adjuvant radiotherapy after breast-conserving surgery, the post-treatment free HSP70 trajectory carries different prognostic weight. Patients in whom serum HSP70 remained persistently elevated in the follow-up period were significantly more likely to relapse (clinical study, *n* = 40 patients) than those in whom levels declined (*p* = 0.03–0.007), with the highest discriminative value achieved at 3–6 months post-RT [149]. Crucially, NK cell counts fell in parallel with the rising HSP70 in patients who subsequently relapsed, a pattern consistent with the interpretation that persistent eHSP70 in the post-RT setting reflects viable residual or recurrent mHSP70-positive tumor cells actively suppressing NK cell function through the vesicular HSP70-TLR2-MDSC axis rather than through residual immunogenic debris from treatment [96,149]. This interpretation aligns with the pre-surgery data from the NSCLC thoracic cancer cohort, where patients with elevated vesicle-associated eHSP70 before resection had significantly higher rates of early relapse after complete resection than those with low pre-surgery levels (626.3 vs. 47.5 ng/mL; *p* < 0.001) (clinical study, *n* = 178 NSCLC patients), independent of tumor stage and histology [10]. Together, these clinical datasets frame a coherent picture in which the vesicle-associated eHSP70 fraction—reflecting viable, mHSP70-positive, NK-suppressive tumor cells—can predict therapeutic failure and recurrence risk, not the total soluble eHSP70 that partly reflects treatment response.

As discussed in Section 3.1.3, pre-treatment vesicular eHSP70 measured by lipHSP70 ELISA correlated positively with GTV in NSCLC [40], indicating that longitudinal eHSP70 monitoring during RT can, under appropriate assay conditions, approximate tumor volume regression non-invasively.

#### 5.2.2. Chemotherapy

Cytotoxic chemotherapy disturbs the eHSP compartment through simultaneously productive and counterproductive mechanisms. On the productive side, effective cytotoxic drugs induce immunogenic tumor cell death, releasing free-soluble HSP70 that activates DCs via TLR4 and potentially augments adaptive anti-tumor immunity, a process analogous to what happens with high-dose RT [96]. On the counterproductive side, sublethal chemotherapy stress on surviving cells upregulates mHSP70 expression, increases exosomal HSP70 secretion, and thereby paradoxically amplifies the very MDSC-activating signal that suppresses anti-tumor T cell responses and protects residual disease [60,96,99].

The eHSP70-mediated macrophage re-education described in Section 4.1.3 is another chemotherapy-specific complication. As demonstrated in vitro and validated in 116 breast carcinoma clinical tissue specimens [99] chemotherapy-induced eHSP70 release from breast cancer cells stimulates TGF-β secretion from the same cells, which in turn polarizes tumor-associated macrophages toward an M2 phenotype characterized by CD163, IL-10, and MMP2 upregulation, a state that supports tumor cell proliferation, chemotherapy resistance, and migration [100]. This circuit creates a form of treatment-induced acquired resistance that is mechanistically distinct from the classical drug efflux or DNA repair resistance pathways: it is an eHSP-mediated immunological adaptation that begins with the drug itself and is amplified by repeated treatment cycles. The clinical evidence consistent with this mechanism is the association of HSP70 tissue expression with macrophage infiltration patterns in breast carcinoma, and the fact that HSP70 neutralization in conditioned medium experiments reverses M2 polarization [100].

The perioperative eHSP70 monitoring data from the surgical oncology setting extend the chemotherapy picture to the neoadjuvant context. In clinical study in thoracic cancer patients receiving neoadjuvant treatment followed by resection, serial eHSP70 measurements distinguished good responders (in whom levels fell progressively) from non-responders (in whom they remained elevated or rose), with the trajectory predicting pathological response ahead of conventional imaging restaging [150]. This parallels what was established for the compHSP70 ELISA in the RT setting and suggests that the same monitoring logic using the vesicle-associated eHSP70 fraction as a real-time indicator of viable chemosensitive vs. chemoresistant tumor mass is transferable across treatment modalities.

#### 5.2.3. Targeted Therapies

The eHSP response to molecularly targeted agents introduces a new dimension not present in cytotoxic or radiotherapy settings: the pharmacological relationship between HSP90 and HSP70 means that drugs targeting oncogenic signaling through HSP90 inhibition inevitably and predictably elevate circulating HSP70 as a direct consequence of their mechanism of action. When HSP90 is inhibited by any of its clinically tested inhibitors (e.g., geldanamycin derivatives (17-AAG, 17-DMAG), resorcinol-based compounds (NVP-AUY922, ganetespib), or purine-scaffold agents (PU-H71)) the chaperone complex that normally sequesters HSF1 in the cytoplasm is disrupted, and HSF1 is derepressed, translocates to the nucleus, and drives robust transcriptional upregulation of both HSP70 and HSP27 [72,151]. The resulting surge in circulating HSP70 in patient peripheral blood mononuclear cells (PBMCs) has been deployed as a pharmacodynamic (PD) biomarker of target engagement across multiple phase I and II clinical trials of HSP90 inhibitors, confirming that pharmacologically active drug concentrations have been achieved at the molecular target [72]. In the combination of KW-2478 and bortezomib in multiple myeloma, this on-target HSP70 induction was synergistically amplified and was used specifically as a PD marker to distinguish the dual-drug effect from either agent alone, with HSP70B (HSPH1) serving as a combination specific readout [73].

What this PD biomarker application reveals about HSP90 inhibitor biology is, however, more nuanced than a simple confirmation of drug exposure. The compensatory HSP70 upregulation induced by HSP90 inhibition is not a neutral indicator of target engagement but is a functionally significant cytoprotective response that partially counteracts the pro-apoptotic consequences of client protein degradation [151]. In in vitro preclinical models with multiple tumor cell lines and non-malignant cells, the radiosensitizing effect of HSP90 inhibitors (17-AAG, geldanamycin, radicicol, NVP-AUY922) was present only in cell cultures where treatment produced early and pronounced HSP70 induction; moreover, the magnitude of radiosensitization was directly proportional to the level of HSP70 induction, not inversely, as might be assumed if HSP70 were purely protective [151]. The mechanistic explanation is that in radiosensitization, HSP70 upregulation functions primarily as a biomarker of the extent of client protein destabilization, rather than as a protective response capable of fully offsetting it at the doses used. Adding an inhibitor of HSP70 induction (quercetin, triptolide, KNK437) to the HSP90 inhibitor prevented HSP70 upregulation and further increased post-radiation cell death in cancer cells without proportionally sensitizing normal cells, supporting the concept that dual HSP90 + HSP70 blockade represents a more complete radiosensitization strategy than either agent alone [151]. The circulating HSP70 level in patients treated with HSP90 inhibitors therefore carries information simultaneously about pharmacological target engagement and about the adequacy of the compensatory heat shock response, a dual readout that, if properly applied with the correct assay, could inform real-time dose adjustments.

#### 5.2.4. Immunotherapy

The intersection of eHSPs and immune checkpoint inhibition is the most therapeutically consequential relationship in this entire section, and also the most recently defined. Two lines of evidence converge to establish eHSP70 as a relevant variable in the biology of anti-PD-1/PD-L1 therapy: the epidemiological association between circulating eHSP90α and clinical outcomes under checkpoint blockade, and the mechanistic role of tumor-intrinsic NLRP3-driven HSP70 secretion in generating adaptive resistance to these agents.

The prognostic correlation was established in a cohort of lung cancer patients receiving the combination of PD-1/PD-L1 inhibitors with chemotherapy, where elevated plasma HSP90α correlated with significantly shorter progression-free and overall survival, independently of PD-L1 expression status [48]. This association is consistent with the biology described throughout Section 5.1 and Section 5.2: high circulating eHSP90α reflects a hypoxic, invasion-competent tumor microenvironment, while a TME that is also MDSC-rich confers resistance to checkpoint blockades. The correlation between pre-treatment eHSP90α and response to immunotherapy is therefore not coincidental but mechanistically grounded. A related finding from the NSCLC cohort characterized by eHSP70 showed that PD-L1-negative tumors had significantly higher vesicle-associated eHSP70 levels than PD-L1-positive tumors (277.7 vs. 21.2 ng/mL), which is itself a readout of the immunological landscape [10]: PD-L1-negative tumors are enriched in mHSP70-positive cells that signal through the vesicular eHSP70-MDSC axis rather than through the adaptive PD-L1-T cell axis, suggesting that eHSP70 and PD-L1 status may be informative in opposite directions about the dominant immune evasion mechanism in any given tumor.

The mechanistic dimension was defined in a series of studies that established the tumor-intrinsic NLRP3 inflammasome as a regulated source of HSP70 secretion that drives adaptive resistance to anti-PD-1 therapy. When CD8+ T cells mount a cytotoxic attack against tumor cells during effective anti-PD-1 treatment, IFN-γ released by these T cells upregulates PD-L1 on tumor cells; PD-L1 intracellular signaling through STAT3 and PKR activates the NLRP3 inflammasome, which drives gasdermin-D-mediated secretion of IL-1β, IL-18, and HSP70 demonstrated in vitro and in mouse tumor models [152]. The secreted HSP70 acts on TLR4 of stromal and myeloid cells in the TME, recruiting granulocytic MDSCs (PMN-MDSCs) that suppress the very T cells generating the IFN-γ signal, closing a negative feedback loop that converts initial anti-PD-1 response into acquired resistance [141,153]. This pathway creates a truly adversarial dynamic: the therapeutic efficacy of checkpoint blockade is the trigger for the resistance mechanism, and the tumor uses the immune pressure generated by treatment to build a more immunosuppressive environment. Pharmacological NLRP3 inhibition combined with anti-PD-1 antibody generated a significantly more effective anti-tumor response than anti-PD-1 monotherapy in the autochthonous BRAFV600E mouse melanoma model, validating the pathway as a therapeutic target rather than an epiphenomenon [153].

The eHSP-based vaccine approaches discussed in Section 5.2 represent the opposite pole of the immunotherapy axis, using eHSPs as immunological activators rather than responding to their presence as a resistance signal. The gp96-based autologous tumor vaccine approach improved outcomes specifically in GBM patients with low PD-L1 expression on peripheral myeloid cells [112], which is not merely a patient selection observation but a mechanistic statement: the immunostimulatory eHSP vaccine is most effective precisely when the NLRP3-HSP70-MDSC resistance pathway is least active. This inverse relationship between the effectiveness of eHSP vaccines and the burden of NLRP3-driven HSP70 secretion in the TME suggests a rational combination strategy. NLRP3 inhibition to suppress tumor-intrinsic MDSC recruitment combined with exogenous eHSP-peptide vaccination to amplify the T cell response in the immunological space opened by NLRP3 blockade.

The convergence of all four therapeutic modalities around eHSP biology is striking when viewed together (Table 14). Radiotherapy, chemotherapy, HSP90 inhibitors, and checkpoint immunotherapy each affect eHSP levels through distinct mechanisms, but in each case the clinical interpretation of the eHSP response requires knowledge of the assay used (vesicular vs. free-soluble fraction), the timing of sampling relative to treatment, and the immunological state of the host.

## 6. Limitations and Translational Challenges

The introduction of circulating eHSP into clinical practice is complicated by a number of limitations, despite the available data. Most studies are retrospective or observational, with small patient cohorts [154]. Given the lack of prospective, multidirectional data, it is difficult to establish validated thresholds for clinical use in decision-making. Additionally, it is impossible to assess the predictive value of eHSP, which is only retrospectively associated with certain conditions. Furthermore, it is unclear whether monitoring eHSP levels can change patient treatment strategies. Based on the current evidence, it is possible to form a hypothesis, but it is not yet possible to justify regulatory approval or widespread clinical use. The analytical approaches used in this study are characterized by methodological heterogeneity, which can lead to significant inter-study variability. The use of different types of biomaterials, such as serum, plasma and tissues, introduces pre-analytical variability due to the lack of standardized protocols for sample collection, processing, and storage [155,156]. It is worth noting that different ELISA platforms, such as standard ELISA for total soluble eHsp70, liposomal lipHSP70 ELISA, and vesicle-specific HSP70-exo ELISA, not only provide systematically different absolute values but also measure different protein pools [157]. Total soluble eHSP70 in serum can reflect the passive release of protein from necrotic cells [158]. Mixing these two pools in the same analysis inevitably reduces the specificity of the measurement [10,158]. Therefore, future studies and clinical validation protocols should provide for the mandatory use of dual isolation methods—a combination of ultracentrifugation or affinity capture for the vesicular fraction with standard total pool ELISA—to distinguish the biological source of the circulating signal. This combination of factors explains the negative or uncertain results in some studies: eHSP70 did not show statistically significant differences between patients with early-stage NSCLC and healthy donors [31], eHSP70 levels in stages I–III of CRC did not exceed those of the control group [158], and the diagnostic accuracy of eHSP70 for prostate cancer did not surpass that of standard PSA screening [14]. These observations should not be interpreted as evidence of the biological unsuitability of this class of markers. Rather, they reflect the lack of standardization in methodological approaches and the stage-specific response of biomarkers. Considering the above, it is essential to provide a systematic indication of the type of biomaterial, analyte form, and detection method used in order to ensure a correct comparison between studies. This is the principle that this review follows in the design of all tables presented.

The predominance of associative data over evidence of causal mechanisms is a second fundamental limitation. The described correlations between eHSP expression levels and disease stage, metastatic status, or prognosis are reliably reproduced in various nosologies [11,13,29,158]. However, the presence of a correlation in itself does not allow us to unambiguously determine whether eHSP is a passive reflection of the tumor load and concomitant inflammation or an active participant in the pathological process. This issue is discussed in detail in Section 4.3 [154]. The only exception for which experimental evidence has been found is HP90A in breast cancer. It has been shown that this protein actively promotes lymphangiogenesis through the LRP1-AKT-CXCL8 axis. Neutralization of this protein by antibodies leads to a decrease in the density of lymphatic vessels of the primary tumor and the frequency of metastasis to sentinel lymph nodes [25]. For the remaining described associations, there was no clinical confirmation of the causal role of eHSP. Direct comparative studies of eHSP with established cancer markers remain equally limited. The available data cover only individual nosologies: HSP90α in comparison with CEA and CA153 in breast cancer [25,36], eHSP70 in comparison with CEA and CA19-9 in colorectal cancer [13], and HSP27 in comparison with PSA in prostate cancer [59]. These studies suggest that eHSPs provide complementary rather than competing diagnostic information. There has been no systematic multicenter comparison of eHSP profiles with integrated panels of standard markers within the framework of a single protocol. Finally, a significant omission in the evidence base is the lack of direct comparative eHSP studies with already approved liquid biopsy methods such as circulating tumor DNA (ctDNA) and circulating tumor cells (CTCs), which have been validated in a wide range of oncological diseases [159]. This does not cast doubt on the value of eHSP as a class of biomarkers, but indicates that their place in the diagnostic algorithm of liquid biopsy remains uncertain compared to already proven instruments and requires specially planned comparative studies.

In the context of clinical limitations, the context-dependent bidirectionality of the biological effects of HSPs, discussed in detail in Section 4.3, deserves separate consideration. From a practical point of view, this means that the same level of circulating HSP70 may reflect diametrically opposite biological situations, depending on the type of tumor, the patient’s immune status and the protein compartment. Thus, an increased level of vesicular HSP70 is associated with a favorable prognosis in grade III gliomas due to the activation of natural killer cells (NK cells). At the same time, in glioblastoma, the same signal reflects immunosuppression and indicates an unfavorable outcome [11,32]. The key factors determining the direction of the effect are the tumor microenvironment, the local cytokine profile, and the degree of preservation of the CD4+ cell population, parameters that are usually not taken into account in clinical biomarker studies [101,160]. This significantly complicates the development of universal thresholds for the expression of endogenous shock proteins (eHSPs) and requires that the interpretation of any measurement of eHSP expression be accompanied by an analysis of the patient’s immunological status.

For eHSP biomarkers to move from the research stage to clinical practice, several conditions must be met. First of all, prospective multicenter studies with pre-registered protocols, standardized pre-analytical procedures, and predefined thresholds are needed. This design allows us to obtain evidence of the predictive rather than the retrospective-associative value of the marker [154]. In addition, validation of specific analytical platforms is necessary. As shown in the methodology section of this paper, the standard ELISA of total eHsp70 and vesicle-specific formats measure biologically different protein pools and cannot be interchanged [155,156]. Finally, the most promising direction is not the isolated use of eHSP, but their inclusion in multi-marker panels. These panels can combine eHSP with already known cancer markers, whose complementarity has been partially confirmed in studies [13,25,36,59], or with ctDNA and CTCs as part of a comprehensive liquid biopsy analysis. The data presented in this review provides the necessary evidence base. However, regulatory approval and implementation of eHSP into clinical protocols require their prospective validation.

## 7. Conclusions

Although circulating eHSPs demonstrate reproducible cancer-type-specific profiles and show clinical value as biomarkers of disease stage, metastatic potential, and therapeutic response, their prognostic significance cannot be decoupled from the eHSP compartment measured, the analytical platform used, and the host immune status. The same circulating level may reflect anti-tumor immunity or immune evasion, productive treatment response or viable residual disease, depending on context. Due to these inherent biological and methodological complexities, alongside the current absence of prospective multicenter validation, eHSP measurements remain insufficient as standalone diagnostic and prognostic tools and require integration within multi-marker panels and standardized assay protocols before meaningful clinical implementation.

## Figures and Tables

**Table 1:** Circulating eHSP70 levels in patients with primary brain tumors and healthy controls.

Tumor Entity	WHO Grade	Sample Type	Median eHSP70 (ng/mL)	Healthy Controls (ng/mL)	Source
GBM (IDH-wt)	IV	Plasma	45.4	18.9	[11]
Astrocytoma (IDH-mut)	III	Plasma	27.5	18.9	[11]
Oligodendroglioma (IDH-mut)	III	Plasma	14.8	18.9	[11]
GBM	IV	Serum	3.5	2.6	[11]
Astrocytoma	III	Serum	3.4	2.6	[23]
GBM (with necrosis)	IV	Serum	Elevated	–	[32]
GBM	IV	Plasma/serum	Elevated	–	[33]

Abbreviations: GBM, glioblastoma; IDH-wt, isocitrate dehydrogenase-wildtype; IDH-mut, IDH-mutant.

**Table 6:** eHSP levels and surface expression in leukemia.

eHSP	Disease	Sample Type	Cancer Level	Control Level	Source
cHSP70	AML	Plasma	Median 10.71 ng/mL	4.13 ng/mL	[62]
cHSP70	ALL	Plasma	Median 27.59 ng/mL	4.13 ng/mL	[62]
cHSP70	MDS	Plasma	Median 4.54 ng/mL	4.13 ng/mL	[62]
cHSP70	CML	Plasma	All phases: 4.17 ng/mL	Lower	[63]
mHsp70	AML blasts	BM	75% mHsp70-positive	0% normal BM	[61]

AML, acute myeloid leukemia; ALL, acute lymphoblastic leukemia; MDS, myelodysplastic syndrome; CML, chronic myeloid leukemia; BM, bone marrow.

**Table 7:** HSP expression, circulating markers, and functional roles in lymphoma and multiple myeloma.

eHSP	Disease	Key Findings	Mechanistic Significance	Source
Serum HSP70 + HSP90	DLBCL	Stage-correlated elevation; decline after R-CHOP	Reflects viable tumor burden; HSP70 mediates therapy resistance in survivors	[66]
Surface HSP90β + STIP1 on B cells	cHL + B-NHL	Overexpressed on reactive, non-clonal B cells	Circulating eHSP90 in lymphoma is not purely tumor-derived	[65]
HSP90 in HRS cells (intracellular)	cHL	Stabilizes JAK-STAT3, NF-κB, CD30, MYC	Core oncogenic driver; HSP90 dependency = therapeutic target	[64]
EV/microvesicle counts (tumor-specific)	AML, HL, MM, CLL, NHL	Elevated; CD30+ in HL, CD38+ in MM, CD19+ in NHL	Tumor-antigen-specific EV immunophenotyping	[67]
Exosomal HSP70 (ExoDiag)	Breast + lung	AUC 0.90 for metastasis; inverse with treatment response	Exo-HSP70 > soluble HSP70 for tumor-specific signal	[68]
Intracellular HSP70; exo-HSP70	MM	Stromal adhesion ↑ HSP70; exo HSP70 transfers bortezomib resistance	eHSP70 mediates resistance spread via exosomes	[69,70,71,74]

cHL, classical Hodgkin lymphoma; B-NHL, B-cell non-Hodgkin lymphoma; DLBCL, diffuse large B-cell lymphoma; MM, multiple myeloma; HRS, Hodgkin/Reed–Sternberg; EV, extracellular vesicle; ↑, increased.

**Table 9:** Altered eHSP levels in cancer [20,24,33,85,86].

eHSP Marker	Cancer Type	Patient eHSP Level (ng/mL)	Healthy Control Level (ng/mL)	Key Notes
eHSP90α	Mixed Cancers (Liver, Lung, Breast, etc.)	Median 157.80	Median 31.19	Pan-cancer elevation confirmed.
eHSP90α	Hepatocellular Carcinoma (Liver)	Median 159.9	Median 30.0	Particularly strong diagnostic performance.
eHSP90α	Lung Cancer	Average 220.46	Average 48.0	Significant elevation observed.
eHSP90α	Acute Myeloid Leukemia	Average 295	Average 12.1	One of the highest reported elevations.
eHSP90α	Nasopharyngeal Carcinoma	Average 212	Average 35	Clear distinction from healthy levels.
eHSP90α	Colorectal Cancer	Average 135	Average 44	Consistent elevation in multiple studies.
eHSP90α	Cervical Cancer	Range 80.6–212.8	Range 48.6–89.6	Upper range significantly higher.
eHSP90α	Gastric Cancer	Median 64.3	45.16	Moderate but significant elevation.
eHSP90α	Melanoma	Median 49.76	Median 25.7	Elevated levels correlate with progression.
eHSP90β	Head & Neck Cancer	65.6	23.5	Specific to the HSP90β isoform.
eHSP70	Pancreatic Cancer	1.68	0.04	Dramatic increase observed.
eHSP70	Non-Small Cell Lung Cancer (NSCLC)	494.1	35.1	High levels detected using specialized assay.
eHSP70	Colorectal Cancer (CRC)	38.83	14.88	Significant elevation confirmed.
eHSP70	Endometrial Cancer	26.05	39.04	** Note: ** This study found lower eHSP70 levels in patients, highlighting cancer-type specificity.
eHSP27	Ovarian Cancer	50.17	13.65	Related small HSP (HSP27) also elevated.

**Table 10:** Specific receptor interactions of eHSPs.

Receptor	eHSP Ligand	Role in Resistance
CD91/LRP1	eHSP70, eHSP90	On cancer cells, activates AKT and ERK signaling promoting survival; on immune cells, can lead to tolerance with chronic exposure [81]
TLR2/TLR4	eHSP70, eHSP90	Chronic activation promotes inflammatory, pro-metastatic environment [26]
CD94/NKG2A	mHSP70	Can be immunosuppressive depending on context [81]

**Table 11:** Tumor-promoting mechanisms of extracellular HSPs.

eHSP	Mechanism	Key Effectors	Functional Outcome	Cancer Relevance	Source
eHSP90α	LRP1 → AKT/NF-κB autocrine signaling	LRP1, AKT, ERK	Tumor cell survival; therapy resistance	Elevated in hypoxia; stage-dependent elevation across tumors	[97]
eHSP90α + MMP2	ECM remodeling; invasion	MMP2/9, co-chaperones AHA1/TIMP2	Matrix degradation; invasion; angiogenesis	Inhibition reduces lung/LN metastasis in multiple models	[97,98]
eHSP90α + Clusterin	EMT via LRP1 → Akt/ERK/NF-κB	E-cadherin ↓, N-cadherin ↑, Snail, Slug	Epithelial–mesenchymal transition	Breast cancer invasion and migration	[98]
eHSP90α + EVs	Paracrine NF-κB/CXCL1 in CAFs	IKKα/β, NF-κB	Blood angiogenesis	Hypoxia-driven; blocked by eHsp90 neutralization	[97]
eHSP90α → LECs	Lymphangiogenesis via LRP1 → AKT → CXCL8	LRP1, AKT, CXCL8	Sentinel LN metastasis ×10	Plasma Hsp90α ↑ with N stage in breast cancer	[25]
eHSP70	Autocrine TLR4/RAGE → NF-κB	NF-κB, PI3K/AKT	Tumor survival; proliferation	CRC, GBM, NSCLC: high eHSP70 → poor prognosis	[73]
eHSP70 + eHSP90α	Co-chaperone complex → MMP2 activation	HSP70, HSP40, HOP, p23	Enhanced invasion	HSP70 depletion from CM impairs eHsp90-mediated MMP2 activation	[98]
Exosomal HSP72/70	TLR2/MyD88 → STAT3 in MDSCs → IL-6	TLR2, MyD88, STAT3, Arg-1, iNOS	T cell suppression; immune evasion	Inhibition by amiloride/A8 aptamer enhances chemo efficacy in vivo	[90,96]
eHSP70 + TGF-β	TAM M2 polarization	TGF-β, CD163, IL-10, MMP2	Chemoresistance, proliferation	Chemo-induced; HSP70 antibody reverses M2 polarization	[100]

CAFs, cancer-associated fibroblasts; LECs, lymphatic endothelial cells; LN, lymph node; CM, conditioned medium; MDSCs, myeloid-derived suppressor cells; TAM, tumor-associated macrophage; EMT, epithelial–mesenchymal transition; ↑, increased; ↓, decreased; →, activates/leads to.

**Table 12:** Context-dependent duality of eHSPs.

eHSP and Context	Anti-Tumor	Pro-Tumor
eHSP70	**Immune Activation:** Acts as a “danger signal” (DAMPs), chaperoning tumor antigens to cross-present to dendritic cells (DCs), activating NK cells [23,114].	**Immunosuppression:** Promotes MDSCs and Tregs, inhibits DC maturation, and induces PD-L1 expression. Promotes survival, metastasis, and therapy resistance [23,114].
eHSP90α	**Immune Stimulation:** Can activate DCs and enhance antigen presentation in some contexts [23,90,91,115].	**Pro-Metastatic:** The most established role. Remodels ECM, induces EMT, and promotes invasion, lymphangiogenesis, and metastasis [23,90,91,115].
Cell Death Context	**Immunogenic Cell Death (ICD):** eHSPs released from dying cells are key signals that recruit and activate the immune system [21,116,117,118].	**Necrosis:** Uncontrolled release of eHSPs from necrotic cells can promote a pro-tumor, inflammatory environment [21,116,117,118].
Location	**Cell Surface (mHSP70):** Can act as a recognition marker for NK cell-mediated killing, which is linked to better prognosis in some cancers (e.g., gastric, colon) [55,70].	**Exosome-Bound:** Tumor-derived exosomes carrying eHSPs are potent promoters of invasion, pre-metastatic niche formation, and immune suppression [117,118].

**Table 14:** Therapy-induced changes in eHSP levels and their clinical significance.

Modality	eHSP Change	Biological Source	Clinical Significance	Source
RT (NSCLC, post-RT)	HSP70 ↑ (responders > non-responders)	Dying tumor cells (free-soluble)	Higher = better RT response; immunogenic cell death	[80]
RT (breast cancer, follow-up)	HSP70 persistent ↑ + NK ↓	Viable residual/recurrent mHSP70+ cells	Predicts recurrence (*p* = 0.03–0.007)	[149]
Surgery (NSCLC, pre-op)	eHSP70 ↑↑ (early relapsers 626 vs. 47.5 ng/mL)	Viable aggressive mHSP70+ tumor cells	Pre-surgery level predicts early relapse	[10]
Chemotherapy (cisplatin, 5-FU)	Exosomal HSP70 ↑	Stressed surviving tumor cells	↑ MDSC activation; blunts anti-tumor immunity	[96]
Chemotherapy (breast)	eHSP70 → TGF-β → M2 macrophages	Surviving tumor cells + macrophages	Chemotherapy-induced resistance; reversible by eHsp70 neutralization	[99,100]
HSP90 inhibitors (clinical trials)	PBMC HSP70 ↑ (derepression)	HSF1 activation in all cells	PD biomarker of HSP90 target engagement	[72,151]
HSP90i + RT	HSP70 ↑ = radiosensitization predictor	Cancer cells: extent of client destabilization	Magnitude of HSP70 induction correlates with radiosensitization; dual HSP90 + HSP70 blockade concept	[151]
Anti-PD-1 (melanoma, lung)	Tumor NLRP3 → HSP70 ↑	Tumor-intrinsic NLRP3 inflammasome	Drives PMN-MDSC recruitment → adaptive resistance; NLRP3 inhibition rescues response	[141,153]
Anti-PD-1 + chemo (lung)	eHSP90α ↑ = worse PFS/OS	Viable tumor cells + aggressive TME	Pre-treatment eHsp90α correlates inversely with ICI benefit	[48]
Autologous gp96 vaccine (GBM)	Vaccine-induced immune priming	Tumor-derived gp96–peptide complexes	Improved OS in PD-L1-low patients; eHSP as therapeutic agent	[112]

PD, pharmacodynamic; compHsp70, complete Hsp70 (free + vesicular); PFS, progression-free survival; OS, overall survival; RT, radiotherapy; ICI, immune checkpoint inhibitor; ↑, increased; ↓, decreased; →, leads to/associated with.

## Data Availability

No new data were created or analyzed in this study.

## Abbreviations

17-AAG	17-allylamino-17-demethoxygeldanamycin
17-DMAG	17-dimethylaminoethylamino-17-demethoxygeldanamycin
ALL	acute lymphoblastic leukemia
AML	acute myeloid leukemia
APC	antigen-presenting cell
AUC	area under the curve
BBB	blood–brain barrier
BCR	biochemical recurrence
BiP	binding immunoglobulin protein
BM	bone marrow
B-NHL	B-cell non-Hodgkin lymphoma
CA19-9	carbohydrate antigen 19-9
CA153	cancer antigen 153
CAF	cancer-associated fibroblast
CEA	carcinoembryonic antigen
cHL	classical Hodgkin lymphoma
CHIP	C-terminus of HSP70-interacting protein
CML	chronic myeloid leukemia
CNS	central nervous system
CRC	colorectal cancer
CSS	cancer-specific survival
CTL	cytotoxic T lymphocyte
CTC	circulating tumor cell
CXCL1	C-X-C motif chemokine ligand 1
CXCL8	C-X-C motif chemokine ligand 8
DAMP	danger-associated molecular pattern
DFS	disease-free survival
DLBCL	diffuse large B-cell lymphoma
ECM	extracellular matrix
EGFR	epidermal growth factor receptor
eHSP	extracellular heat shock protein
EMT	epithelial-to-mesenchymal transition
EpCAM	epithelial cell adhesion molecule
EphA2	ephrin type-A receptor 2
ERG	ETS-related gene
ERK	extracellular signal-regulated kinase
EV	extracellular vesicle
FDA	US Food and Drug Administration
GBM	glioblastoma multiforme
GJIC	gap-junction intercellular communication
gp96	glycoprotein 96
GRP75	75 kDa glucose-regulated protein
GRP78	78 kDa glucose-regulated protein
GRP94	94 kDa glucose-regulated protein
GTV	gross tumor volume
HIF-1α	hypoxia-inducible factor 1 alpha
HL	Hodgkin lymphoma
HRS	Hodgkin/Reed-Sternberg
HSC70	heat shock cognate 70 kDa protein
HSF1	heat shock factor 1
HSP	heat shock protein
HSP27	heat shock protein 27
HSP40	heat shock protein 40
HSP60	heat shock protein 60
HSP70	heat shock protein 70
HSP72	heat shock protein 72
HSP90	heat shock protein 90
HSP90α	heat shock protein 90 alpha
HSP90AA1	heat shock protein 90 alpha family class A member 1
HSP90β	heat shock protein 90 beta
HSPA1A	heat shock protein family A member 1A
HSPA1B	heat shock protein family A member 1B
HSPA1L	heat shock protein family A member 1L
HSPA5	heat shock protein family A member 5
HSPA9	heat shock protein family A member 9
HSPB1	heat shock protein beta-1
HSPH1	heat shock protein family H member 1
ICI	immune checkpoint inhibitor
IDH	isocitrate dehydrogenase
IFN-γ	interferon gamma
IKK	IκB kinase
IL-6	interleukin 6
IL-10	interleukin 10
IL-12	interleukin 12
IL-1β	interleukin 1 beta
IL-18	interleukin 18
LC	lung cancer
LDH	lactate dehydrogenase
LEC	lymphatic endothelial cell
LOX-1	lectin-type oxidized LDL receptor 1
LRP1	low-density lipoprotein receptor-related protein 1
LUAD	lung adenocarcinoma
MAST1	microtubule-associated serine/threonine kinase 1
mCRPC	metastatic castration-resistant prostate cancer
MDSC	myeloid-derived suppressor cell
MDS	myelodysplastic syndrome
MHC	major histocompatibility complex
mHSP70	membrane-bound HSP70
MM	multiple myeloma
MMP2	matrix metalloproteinase 2
MMP9	matrix metalloproteinase 9
MyD88	myeloid differentiation primary response 88
NF-κB	nuclear factor kappa B
NK	natural killer
NLRP3	NLR family pyrin domain containing 3
NS	not significant
NSE	neuron-specific enolase
NSCLC	non-small cell lung cancer
OS	overall survival
PBMC	peripheral blood mononuclear cell
PCa	prostate cancer
PD	pharmacodynamic
PD-1	programmed cell death protein 1
PDAC	pancreatic ductal adenocarcinoma
PD-L1	programmed cell death ligand 1
PFS	progression-free survival
PI3K	phosphatidylinositol 3-kinase
PKR	protein kinase R
PMN-MDSC	polymorphonuclear myeloid-derived suppressor cell
PSA	prostate-specific antigen
R-CHOP	rituximab, cyclophosphamide, doxorubicin, vincristine, prednisone
RAGE	receptor for advanced glycation end-products
RASP	resistance-associated secretory phenotype
RT	radiotherapy
SCLC	small cell lung cancer
sEV	small extracellular vesicle
SREC-1	scavenger receptor expressed by endothelial cells 1
STAT3	signal transducer and activator of transcription 3
STIP1	stress-induced phosphoprotein 1
TAM	tumor-associated macrophage
TGF-β	transforming growth factor beta
TKD	TKDNNLLGRFELSG peptide
TLR	Toll-like receptor
TLR2	Toll-like receptor 2
TLR4	Toll-like receptor 4
TME	tumor microenvironment
TNF-α	tumor necrosis factor alpha
TNM	tumor-node-metastasis
VEGF	vascular endothelial growth factor
WHO	World Health Organization
